# Supplementary material for: Comparison of self-collected vaginal swabs and first-void urine for detection of human papillomavirus in sexually active girls and women in three South Asian countries
Source: PLoS One. 2026 Jun 12;21(6):e0350049. doi: 10.1371/journal.pone.0350049 (PMC13262861; doi:10.1371/journal.pone.0350049)
Supplement: S1 File — (DOCX) [file pone.0350049.s001.docx]

1. **Instruction for Field Staff**

**Step 1**. Welcome the participant and obtain her consent to produce the self-collected vaginal swab (SCVS)

**Step 2**. Give instructions to the participant on how to bring out the swab stick, how to hold the stick, and the swab collection procedure. Demonstrate the collection procedure; **lay emphasis on how to avoid contamination. Let the participant know she can you and get help at any time during the collection process.**

1. **Instruction for Participants**

**3**


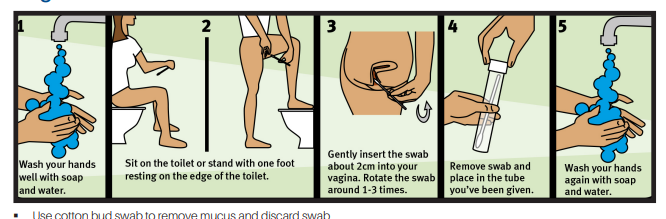


**Step 3**. Wash your hands

or clean them with

hand sanitizer before starting.

**4**


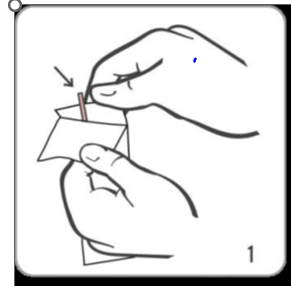


**Step 4**. Peel open the swab package as shown in **Diagram 4**. Remove the swab.

Do not touch the soft tip or lay the swab down. If the soft tip is touched,

the swab is laid down, or the swab is dropped, ask the staff for a new kit.

**Step 5**. Hold the swab in your hand as shown


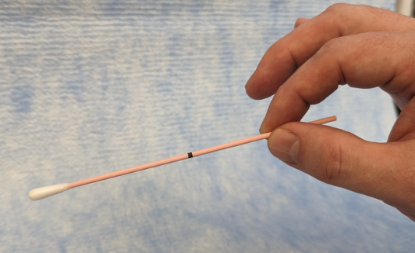


**5a**

**5b**

in **Diagram 5a**, placing your thumb and forefinger

on the swab stick at the opposite end from the soft

tip. Do not hold the swab shaft on or below the

score line (**black line, Diagram 5b**).

**
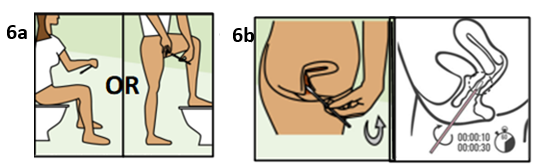
Step 6**. Sit on the toilet or stand with one foot resting on the edge of the toilet (**Diagram 6a**). Carefully insert swab deeply into your vagina, pushing gently until there is some resistance and gently rotate swab clockwise for 10 - 30 seconds or 3 to 5 times (**Diagram 6b**). Then withdraw the swab without touching the skin.

**Step 7.** Call the Field Staff to receive the swab and continue with the process. Wash you hands after handing over the swab to the Filed Staff.

1. **Second Instruction for Field Staff**

**9**


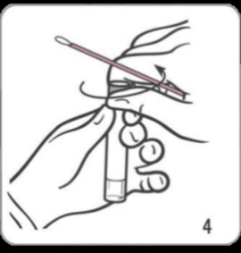


**9**

**Step 8**. Receive the collected swab from the participant

**Step 9**. While holding the swab in the same hand, unscrew the cap from the tube as shown in **Diagram 9. Do not spill the contents of the tube**. If the contents of the tube are spilled, take a new kit and do the collection once more, but explain to the participants.

**10aa**


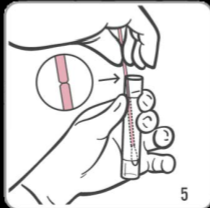

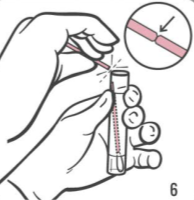


**10ba**

**10ca**

**Step 10**. Immediately place the swab into

the transport tube so that the score line (**black line**)

is at the top of the tube as shown in **Diagram 10a**.

Carefully break the swab shaft at the score line **(black line**)

against the side of the tube as shown in **Diagram 10b**.

Immediately discard the top portion of the swab shaft (**Diagram 10c**).

**11**

**Step 11.** Close the tube tightly and label with barcode stickers appropriately (**Diagram 11**).
