# Supplementary material for: Comparison of self-collected vaginal swabs and first-void urine for detection of human papillomavirus in sexually active girls and women in three South Asian countries
Source: PLoS One. 2026 Jun 12;21(6):e0350049. doi: 10.1371/journal.pone.0350049 (PMC13262861; doi:10.1371/journal.pone.0350049)
Supplement: S2 File — (DOCX) [file pone.0350049.s002.docx]

1. **Instructions for Field Staff:**


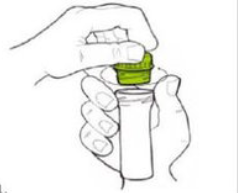


**1**

**Step 1**. Unscrew the cap and carefully place it on the workbench, avoiding contamination.


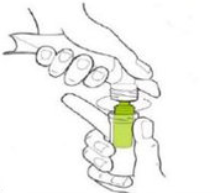


**2**

**Step 2**. Attach or screw/push the collector tube

on the Colli-Pee® housing. Do not touch the bottom

part of the Colli-PeeÂ® (green part)

**Step 3:** Give the assembled colli-pee device to the participant to go and collect urine sample. Inform the participant to bring back the urine with the assembled device as it is.

1. **Instructions for the Participant:**

- Don’t wash your intimate zone before urinating.
- Do not urinate for an hour before collection.
- Do not interrupt the urine stream till the end.

**Step 4**. Collect urine. The device will automatically collect the correct volume.


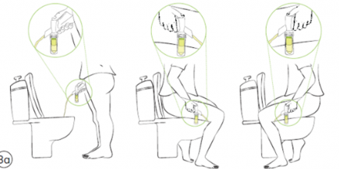


**4**

**Step 5:** return the colli-pee device with the urine sample to the filed staff and wash your hands

1. **Final Instructions for Field Staff:**


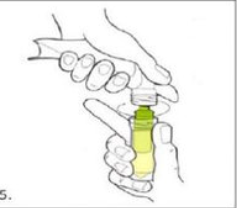


**6**

**Step 6**. Receive the specimen from the participant and carefully disconnect the connector tube

from the Colli-Pee® housing.

**Step 7**. Dispose the Colli-Pee device according to local regulations for plastic waste.


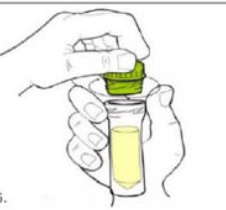


**8**

**Step 8**. Recap the urine tube with its cap; close the tube tightly until it makes a click sound.

**Step 9**. Label the urine specimen with the appropriate barcode sticker and package the specimen for transportation to the laboratory.
