# Supplementary figures and images for: Comparison of self-collected vaginal swabs and first-void urine for detection of human papillomavirus in sexually active girls and women in three South Asian countries
Source: PLoS One. 2026 Jun 12;21(6):e0350049. doi: 10.1371/journal.pone.0350049 (PMC13262861; doi:10.1371/journal.pone.0350049)

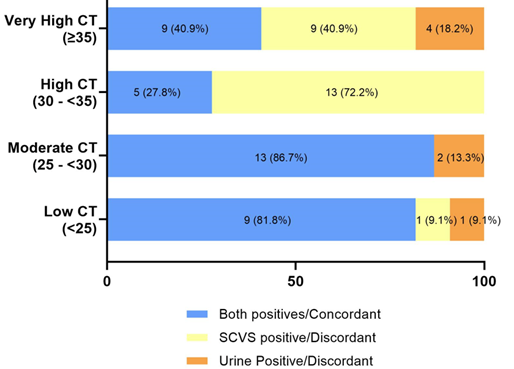

Supplement: S1 Fig — (TIF) [file pone.0350049.s003.tif]

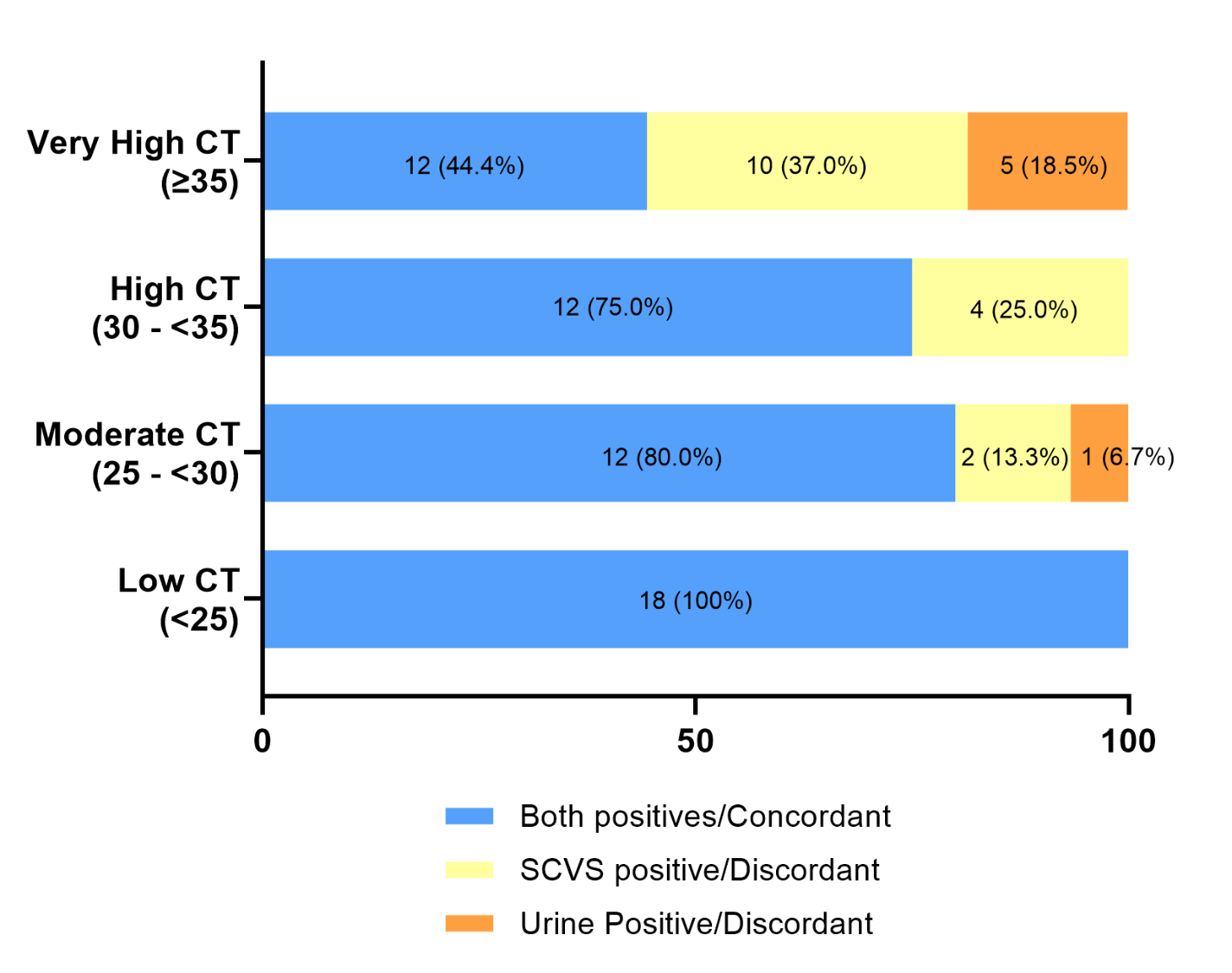

Supplement: S2 Fig — (TIF) [file pone.0350049.s004.tif]

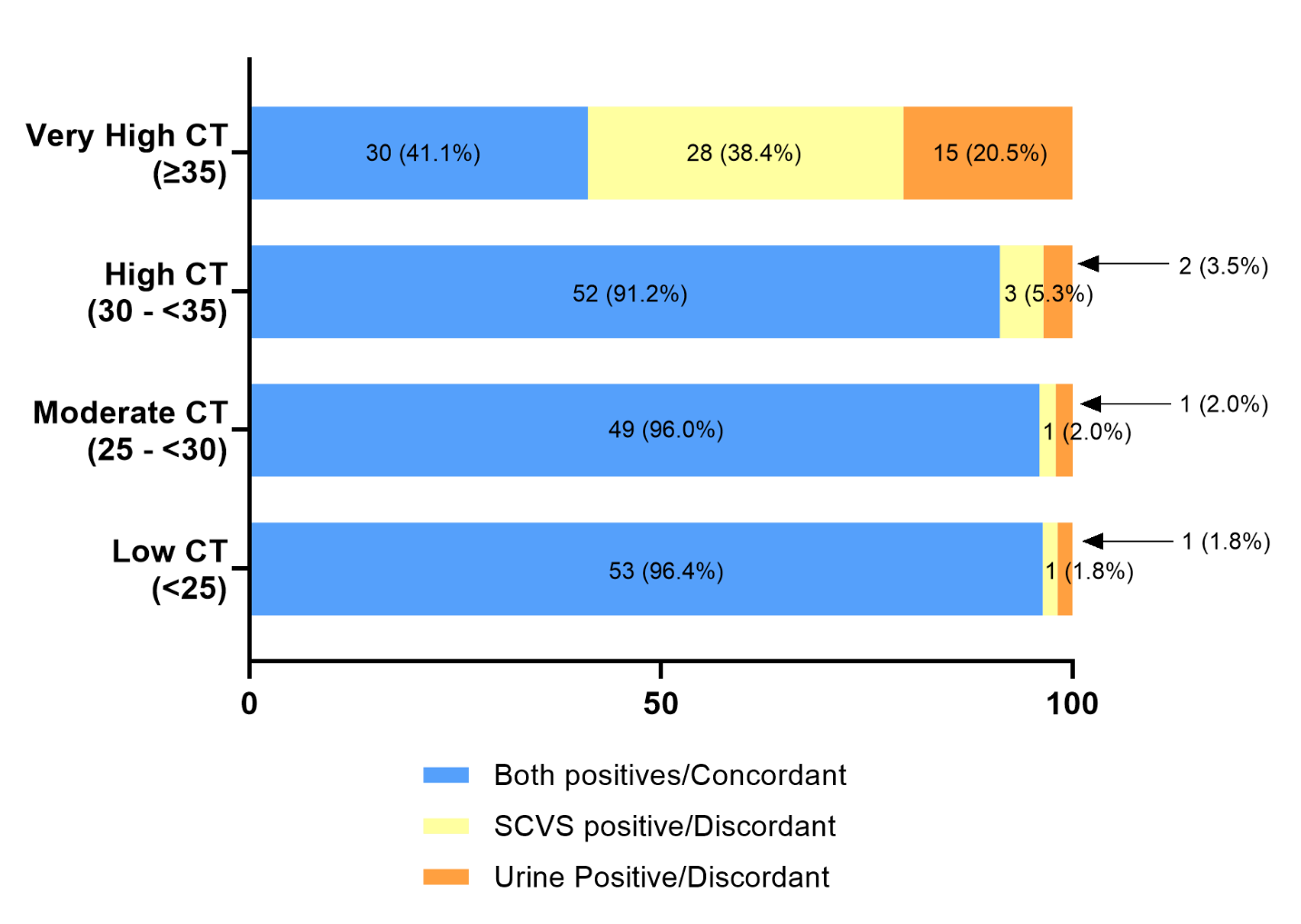

Supplement: S3 Fig — (TIF) [file pone.0350049.s005.tif]
