# Supplementary material for: Comparison of self-collected vaginal swabs and first-void urine for detection of human papillomavirus in sexually active girls and women in three South Asian countries
Source: PLoS One. 2026 Jun 12;21(6):e0350049. doi: 10.1371/journal.pone.0350049 (PMC13262861; doi:10.1371/journal.pone.0350049)
Supplement: Attachment — Submitted filename: report_10.1101+2025.08.01.25332560.pdf [file pone.0350049.s007.pdf]

ScreenIT Report

This is an automated report for 10.1101+2025.08.01.25332560.pdf. This report was solicited by the PLOS One editorial team and provided by ScreenIT.

ScreenIT is an independent group of scientists developing automated tools that analyze academic papers. A set of automated tools screened your submitted manuscript and provided the report below. Each tool was created by your academic colleagues with the goal of helping authors.

The tools look for factors that are important for transparency, rigor and reproducibility, and we hope that the report might help you to improve reporting in your manuscript. Within the report you will find links to more information about the items that the tools check. These links include helpful papers, websites, or videos that explain why the item is important.

While our screening tools aim to improve and maintain quality standards they may, on occasion, miss nuances specific to your study type or flag something incorrectly. Each tool has limitations that are described on the ScreenIT website. The tools screen the main file for the paper; they are not able to screen supplements stored in separate files.

Please note that the Academic Editor had access to these comments while making a decision on your manuscript. The Academic Editor may ask that issues flagged in this report be addressed.

If you would like to provide feedback on the ScreenIT tool, please email the team at ScreenIt@bih-charite.de. If you have questions or concerns about the review process, please contact the PLOS One office at plosone@plos.org.

How to read the report:

- no action required

? - review for potential issues

! - a critical issue was found by a tool

n/a - may not be applicable to your paper

- more information about this item can be obtained using this link

Rigor

✓	Ethics	Consent: "When eligible for the longitudinal cohort girls/women provided informed consent or assent to provide samples every 6 months over the course of 2 years."<br>Field Sample Permit: "Sample collection and test procedure This sub-study was conducted under a harmonized sampling and laboratory protocol to ensure comparability of results from participating"
✓	Inclusion and Exclusion Criteria	"Inclusion and exclusion criteria Girls and women were deemed eligible if they met the following criteria: aged within the target range, sexually active, residents of the study community for at least the past 3 months, and able to understand the purpose of the study and study procedures."
?	Flow charts and attrition	We did not find a study flow chart of excluded observations. We strongly recommend using flow charts because they provide an overview of the study design and more information about attrition. If you included a study flow chart in your supplemental files, we apologize for missing this. Our tool is not able to screen separate supplemental files.<br><br>Sentence about attrition: "One of its particular aims is to assess the incidence of persistent HPV infection through longitudinal cohort studies among sexually active girls and young women (38)."
✓	Sex as a biological variable	"Materials and Methods Study design The Global Burden Estimation of HPV (GLOBE-HPV) study is a multi-country, multi-site project aimed at estimating the burden of HPV among girls and women in 8 GAVI-eligible countries in South Asia (Bangladesh, Pakistan, and Nepal) and sub-Saharan Africa (Democratic Republic of Congo, Ghana, Sierra Leone, Tanzania, and Zambia)."
✓	Subject Demographics	Age: "The study enrolled 250 ever-married women (25 years old) through stratified systematic sampling."

?	Randomization	Not detected. If you performed an experimental study, please specify whether participants, animals or samples were randomly assigned to treatments or groups, and specify the randomization procedure.
?	Blinding	Not detected. Please specify whether blinding was used at various phases of the experiment (e.g., blinding of patients, caregivers, outcome assessments, data analysis).
?	Power Analysis	Not detected. Please state how you determined the sample size for the study. If you performed a power or sample size calculation, please provide details.
n/a	Replication	not required.

Generated by Barzooka (flow charts), SciScore (everything else). SciScore only screens the text of the methods section; we apologize if the tool has missed items reported elsewhere in the paper or supplements.

Resources

Persistent unique identifiers for research resources, RRIDs, can be used to track these key resources though the literature. They are requested or required in many journals, but even if your journal does not require them, we strongly encourage the use of these for the readers of papers who wish to easily track down the key materials like antibodies, software tools, or mice.

Software and Algorithms	"Results were analyzed using Seegene Viewer and formatted for REDCap."	? REDCap suggested: REDCap (RRID:SCR_003445). Please make sure this RRID suggestion is accurate before including in your manuscript.
Software and Algorithms	"Data entry, processing and statistical analysis All data were entered into REDCap, and analyses conducted using SAS 9.4 (SAS	? SAS Institute suggested: Statistical Analysis System, RRID:SCR_008567. Please make sure this RRID suggestion is accurate before including in your manuscript.

	Institute, Cary, NC) and Stata 18"	
Software and Algorithms	"(StataCorp LLC, College Station, TX)."	StataCorp suggested: Stata, RRID:SCR_012763. Please make sure this RRID suggestion is accurate before including in your manuscript.

Generated by SciScore.
SciScore only screens the text of the methods section; we apologize if the tool has missed items reported elsewhere in the paper or supplements.

Transparency

?	Open code	If you wrote code to analyze your data, please consider sharing the code in a public repository. This makes it easier for others to reproduce your analyses, and may also aid others seeking to analyze similar datasets.
?	Open data	If permitted, sharing data on a public repository with a persistent identifier (a DOI or accession number) can improve reproducibility and make it easier for other scientists to expand on your work. Papers with open data are cited more often than papers without open data. Some institutions have an expert who can provide advice on data sharing.
?	Self-acknowledged limitations	An explicit section about the limitations of this study was not found. We encourage authors to include a paragraph in the discussion that addresses study limitations. Every study has limitations. Describing these limitations helps readers to understand and contextualize the research.
?	Conflict of interest statement	It is important to supply a conflict of interest statement, even if there are none to declare, to transparently present the research.
?	Funding statement	It is important to supply a funding statement because it makes the funding source and potential conflicts of interests transparent.
n/a	Registration statement	A registered protocol makes the planned research project more transparent and can protect against bias. Please consider registering future confirmatory studies.

Generated by ODDPub, limitation-recognizer, rtransparent, trial-identifier.

Figures

	No bar graphs of continuous data	Thank you for not using bar graphs to present continuous data. Many different datasets and data distributions can lead to the same bar graph, and the actual data may suggest different conclusions from the summary statistics alone.
	No rainbow color maps	Thank you for not using rainbow colormaps. Rainbow color maps distort readers’ perception of the data by introducing visual artifacts and are not accessible to colorblind readers.

Generated by JetFighter, Barzooka.
