# Supplementary material for: Comparison of self-collected vaginal swabs and first-void urine for detection of human papillomavirus in sexually active girls and women in three South Asian countries
Source: PLoS One. 2026 Jun 12;21(6):e0350049. doi: 10.1371/journal.pone.0350049 (PMC13262861; doi:10.1371/journal.pone.0350049)
Supplement: Attachment — Submitted filename: Comments PLoS One.docx [file pone.0350049.s006.docx]

The article entitled, “Comparison of self-collected vaginal swabs and first-void urine for detection of human papillomavirus in sexually active girls and women in three South Asian countries,” by Telele et al., describes a cross-sectional study wherein the authors compared two non-invasive sampling methods for HPV DNA detection. The authors collected 753 samples from sexually active females ages 15–25 in Bangladesh, Pakistan, and Nepal using two methods, i.e., self-collected vaginal swabs (SCVS) and first-void urine (FVU). The authors used a harmonized protocol not only to assess HPV prevalence but also to determine concordance between the two sample types.

Though the study appears to be scientifically sound, and the manuscript is well organized and prepared, there are few changes required to strengthen the objectives and outcomes of the study. The manuscript can be accepted for publication in the PLoS One after replies to the following comments are addressed satisfactorily:

1.	The authors cited similar studies that have compared self-collected vs urine samples for HPV. The authors should mention with clarity what is the uniqueness/novelty of the present study.

2.	In the introduction section, instead of over emphasizing the HPV background and self-sampling the authors should focus on the research question and what knowledge gap is being filled by carrying out the present study.

3.	The authors should provide the justification of cohort chosen especially the reason for age of 15-25 years and if / how it is related to HPV vaccination. Why do authors have not included broader age range? The reasoning behind the narrative should be provided in the manuscript as it would strengthen the manuscript.

4.	The recruitment strategies detailed in the present study differed by country. Such as, the authors carried clinical based sampling in Nepal; demographic surveillance in Pakistan and sampling of married women in Bangladesh. The impact of recruitment strategy can be on the results / observations of the study. The authors should provide a clarification on the approach.

5.	In discussion section, the authors could provide information on comparing the practical advantages between the two methods of sampling. They should also discuss how the present study outcomes can have influence on screening strategies. The authors can summarize the major findings/takeaways of the present study in conclusion.

6.	As the samples were self-collected, the authors should acknowledge the limitations of the method because of absence of clinician / trained personnel collected samples as it can impact the outcome of the result. The authors should mention other limitations too.

7.	The authors should provide the probable reasons for the observations that why Nepal had higher HPV prevalence than other two countries. Further, authors should mention if they have any plans for future studies to follow up or some other objectives.

8.	The authors should ensure consistency in use of HPV-related terms. Such as at some places for High Risk ‘hr’ is used while at other places ‘HR’ is used. Authors should check the manuscript to make sure about uniformity.

9.	Authors should make sure that all web references have accessed date and formatted as per PLoS One requirements. Also, make sure that all the references are cited and citations provided meet the PLoS One requirements.
