# Supplementary material for: Comparison of self-collected vaginal swabs and first-void urine for detection of human papillomavirus in sexually active girls and women in three South Asian countries
Source: PLoS One. 2026 Jun 12;21(6):e0350049. doi: 10.1371/journal.pone.0350049 (PMC13262861; doi:10.1371/journal.pone.0350049)
Supplement: Attachment — Submitted filename: General Comments.docx [file pone.0350049.s008.docx]

General Comments:

- This manuscript reports original, multicountry research comparing self-collected vaginal swabs (SCVS) and first-void urine (FVU) for HPV detection among young women in South Asia. The study addresses an important public health question in low- and middle-income countries and contributes valuable evidence for HPV surveillance and screening strategies.

- Minor language editing could further enhance readability.

Abstract

- The abstract is well structured and clearly summarizes the background, objectives, methods, key findings, and conclusions. The study design, sample size, laboratory methods, and main statistical approaches are appropriately described. The results are presented quantitatively and support the stated conclusions. The abstract accurately reflects the content of the manuscript and meets PLOS ONE standards for clarity and completeness

Introduction

- Provides a comprehensive overview of HPV epidemiology, cervical cancer burden, and screening challenges in South Asia. The authors effectively contextualize the need for self-sampling approaches and cite relevant international and regional literature. The rationale for comparing SCVS and FVU is well justified, and the research objectives are clearly stated.

- Lines 44–60 in the Introduction contain detailed country-specific descriptions of cervical cancer burden in Bangladesh, Pakistan, and Nepal. While this information is relevant, this section could be streamlined to improve conciseness and flow. Given that the study focuses on three countries, the authors may consider briefly explaining the rationale for selecting these specific settings (e.g., representativeness, programmatic relevance, epidemiological burden, or participation in the GLOBE-HPV project). In addition, the epidemiological background could be summarized more succinctly by presenting key indicators—such as HPV prevalence, cervical cancer incidence, and mortality rates—for each country, rather than providing extended narrative descriptions. Condensing this section would enhance readability while maintaining its contextual value, and would allow readers to more quickly understand the public health relevance of the selected study sites.

Materials and Methods

The manuscript states that in Bangladesh and Pakistan, participants were required to be married, whereas in Nepal eligibility was based on being sexually active regardless of marital status.

Could the authors please clarify the rationale for this difference in inclusion criteria across countries?

Specifically:

- Was marital status used as a proxy for sexual activity in Bangladesh and Pakistan due to sociocultural or ethical considerations?

- Were there regulatory or institutional constraints that prevented recruitment of unmarried sexually active women in those settings?

- How might this difference affect comparability of HPV prevalence and concordance estimates across the three countries?

A brief explanation in the Methods section, and possibly a note in the Limitations section addressing potential selection bias or differences in sexual behavior patterns, would strengthen transparency and interpretation of cross-country comparisons.

Sample Size

Could the authors please clarify:

- Whether a formal sample size or power calculation was performed for this concordance analysis?

- If so, which assumptions were used (expected HPV prevalence, anticipated agreement/Kappa value, precision, alpha level)?

- Whether the target of approximately 250 participants per country was based on logistical considerations, cohort design, or statistical requirements of the GLOBE-HPV project?

Results

- The manuscript reports no inadequate test results; however, additional clarification on data completeness (e.g., missing demographic or behavioral variables) would improve transparency.

- The description of discordant cases could be expanded, for example by indicating whether discordance was more frequent in specific subgroups (country, age range, or HPV type categories).

Discussion

The Discussion section is generally well written and provides an appropriate interpretation of the study findings in relation to existing literature. The authors successfully contextualize their results within prior studies on self-sampling for HPV detection and highlight the relevance for surveillance in low- and middle-income settings.

However, several aspects could be strengthened:

**- Balance between interpretation and speculation** Some explanations regarding differences in sensitivity and concordance between SCVS and FVU appear speculative and would benefit from stronger empirical support or clearer citation of supporting evidence.

**- Cross-country comparability** Given the differences in inclusion criteria and recruitment strategies across countries, the authors should more explicitly discuss how these variations may influence the comparability of findings and interpretation of pooled analyses.

**- Public health and implementation implications** While the potential use of SCVS and FVU in surveillance programs is mentioned, further discussion on operational feasibility, cost considerations, and integration into existing screening systems in South Asia would enhance the practical relevance of the findings.

**- Limitations and potential biases** The limitations section could be expanded to more clearly address potential selection bias, reporting bias related to self-sampling, and variability in laboratory processing across sites.
