# Supplementary material for: Comparison of self-collected vaginal swabs and first-void urine for detection of human papillomavirus in sexually active girls and women in three South Asian countries
Source: PLoS One. 2026 Jun 12;21(6):e0350049. doi: 10.1371/journal.pone.0350049 (PMC13262861; doi:10.1371/journal.pone.0350049)
Supplement: Attachment — Submitted filename: Response to Reviewers.docx [file pone.0350049.s009.docx]

**Authors’ response to the technical comments and reviewers’ comments:**

Dear Editor,

**“Comparison of self-collected vaginal swabs and first-void urine for detection of human papillomavirus in sexually active girls and women in three South Asian countries” (PONE-D-25-43925)**

Thank you for the opportunity to revise our manuscript. We have addressed the reviewers’ comments point by point and made revisions when needed. Locations of changes are noted using page and line numbers in the manuscript with track-change version. At the end, we have provided responses to the comments received with MS-Word file attachments.

We hope that the revised version of this manuscript is now suitable for publication in PLOS One.

**Response to the technical comments:**

Reviewer #1

The article entitled, “Comparison of self-collected vaginal swabs and first-void urine for detection of human papillomavirus in sexually active girls and women in three South Asian countries,” by Telele et al., describes a cross-sectional study wherein the authors compared two non-invasive sampling methods for HPV DNA detection. The authors collected 753 samples from sexually active females ages 15–25 in Bangladesh, Pakistan, and Nepal using two methods, i.e., self-collected vaginal swabs (SCVS) and first-void urine (FVU). The authors used a harmonized protocol not only to assess HPV prevalence but also to determine concordance between the two sample types.

Though the study appears to be scientifically sound, and the manuscript is well organized and prepared, there are few changes required to strengthen the objectives and outcomes of the study. The manuscript can be accepted for publication in the PLoS One after replies to the following comments are addressed satisfactorily:

1.	The authors cited similar studies that have compared self-collected vs urine samples for HPV. The authors should mention with clarity what is the uniqueness/novelty of the present study.

**Response: While self-collected swabs and urine samples have been studied as methods for HPV detection, the methods used, including collection devices, preservatives, processing, extraction and assays all differ. Our results add to options that have been evaluated and include the ability to perform the collection and testing in a harmonized manner. Further, we included an exploration of the impact of viral load on the concordance between samples. We modified the introduction to clarify this (page 6, lines 98 – 101).**

2.	In the introduction section, instead of over emphasizing the HPV background and self-sampling the authors should focus on the research question and what knowledge gap is being filled by carrying out the present study.

**Response: The introduction has been extensively modified to emphasize the knowledge gap being addressed.**

3.	The authors should provide the justification of cohort chosen especially the reason for age of 15-25 years and if / how it is related to HPV vaccination. Why do authors have not included broader age range? The reasoning behind the narrative should be provided in the manuscript as it would strengthen the manuscript.

**Response: The cohorts were formed based on the aims of the GLOBE-HPV study. The age group for the comparison of sampling methods was selected primarily because of the likelihood of HPV acquisition. In addition, this age group is generally considered for “catch-up” vaccination, so providing an indication of exposure to vaccine types could be of importance to countries planning HPV vaccine campaigns.**

4.	The recruitment strategies detailed in the present study differed by country. Such as, the authors carried clinical based sampling in Nepal; demographic surveillance in Pakistan and sampling of married women in Bangladesh. The impact of recruitment strategy can be on the results / observations of the study. The authors should provide a clarification on the approach.

**Response: Marital status was used as a proxy for sexual activity in Bangladesh and Pakistan due to sociocultural, religious, or ethical considerations. Asking about sexual activity outside of marriage is culturally sensitive and could cause repercussions for the participants. This explanation has been added. We do not think that this difference in recruitment impacts study findings as the cultures of Bangladesh and Pakistan result in near concordance between sexually active women and married women (page 9, lines 153 -156).**

5.	In discussion section, the authors could provide information on comparing the practical advantages between the two methods of sampling. They should also discuss how the present study outcomes can have influence on screening strategies. The authors can summarize the major findings/takeaways of the present study in conclusion.

**Response: The major takeaway of the present study is included in the conclusion, that is, both sampling methods give similar HR-HPV results. We did not collect data on practical advantages between the two methods. These would include preference of the women (not addressed), costs of materials and disposal (different in each setting due to local importing suppliers and tariffs), and laboratory facilities (standardized and key assay instrument provided as part of GLOBE-HPV).**

6.	As the samples were self-collected, the authors should acknowledge the limitations of the method because of absence of clinician / trained personnel collected samples as it can impact the outcome of the result. The authors should mention other limitations too.

**Response: The biggest limitation to the findings is that they apply to the specific sample collection tools, media, processing, extraction and assays used in this study and may not be generalizable to all methods. Lack of clinician collected samples as comparator was added to the limitations and the potential impact of population-specific factors and laboratory variations was added (page 20, lines 364 – 374).**

7.	The authors should provide the probable reasons for the observations that why Nepal had higher HPV prevalence than other two countries. Further, authors should mention if they have any plans for future studies to follow up or some other objectives.

**Response: This study is focused on comparability of sampling methods. Discussion of country-specific differences in HPV prevalence as well as persistence will be addressed in the GLOBE-HPV publications.**

8.	The authors should ensure consistency in use of HPV-related terms. Such as at some places for High Risk ‘hr’ is used while at other places ‘HR’ is used. Authors should check the manuscript to make sure about uniformity.

**Response: Checked.**

9.	Authors should make sure that all web references have accessed date and formatted as per PLoS One requirements. Also, make sure that all the references are cited and citations provided meet the PLoS One requirements.

**Response: Checked**

Reviewer #4:

This manuscript presents a valuable multi-country study evaluating the concordance between self-collected vaginal swabs (SCVS) and first-void urine (FVU) for HPV detection among young women in Bangladesh, Nepal, and Pakistan. The study addresses a critical public health need in resource-limited settings. The methodology is robust, the sample size is adequate, and the manuscript is well-written. The harmonized protocol across three countries is a significant strength.
However, I have a few minor comments and queries that, if addressed, would further strengthen the manuscript.
Specific Comments:
Sample Validity: The results state (Page 12, Line 196) that "No inadequate test results were reported from either sample type." Given the nature of self-collection and the large sample size (N=753 pairs), a 0% invalid rate is exceptionally evident of high-quality supervision or robust DNA extraction methods. Could the authors briefly clarify in the Methods section if any participants were excluded prior to the final N=753 count due to sample insufficiency, or if the "UCM" buffer and extraction protocol truly yielded 100% valid DNA for all collected samples? This detail speaks to the feasibility of this method in real-world surveillance.

**Response: None of the samples were inadequate in this set of samples, although this has been encountered in the larger study. This information has been added to the results (page 13, lines 227).**

Data Availability: The authors have indicated that data is available "upon reasonable request" due to ethical restrictions. Please ensure this complies with PLOS ONE’s strict data availability policy. If the data contains sensitive patient information that prevents public deposition, the specific ethical restrictions and the contact information for a non-author institutional body (e.g., an Ethics Committee) should be clearly stated in the final declaration, rather than just the authors' contact.

**Response:** **De-identified individual participant data underlying the results reported in this article will be made available through Vivli (vivli.org), where access requires submission and approval of a formal data access request. This information has been added (page 1, lines 31-33).**

Cycle Threshold (Ct) Analysis: The comparison of Ct values between concordant and discordant samples is a very insightful addition (Figure 1). It clearly demonstrates that discordance is driven by low viral loads. It would be beneficial to briefly discuss the clinical implication of this: are the "missed" infections in discordant cases likely to be clinically significant (e.g., persistent infections) or transient low-load infections?

**Response:** **We do not have data to address the clinical significance of low viral load infections. While it is tempting to speculate that these are less likely to persist and progress, the study was not designed to address this question.**

Demographics: The study population varies slightly by country (e.g., married women in Bangladesh/Pakistan vs. sexually active women in Nepal). While the authors acknowledge this in the limitations, a brief sentence in the Discussion on whether marital status (and implied sexual behavior differences) might impact the vaginal microbiome or viral load—and thus detection concordance—would be interesting, though not mandatory.

**Response: These cohorts were intended to represent sexually active women, however asking about sexual activity outside of marriage in Bangladesh and Pakistan was not permissible due to sociocultural, religious, or ethical considerations. Marriage was used as a proxy for sexual activity. We do not think that this difference in recruitment impacts study findings as the cultures of Bangladesh and Pakistan result in near concordance between sexually active women and married women. The concordance findings between countries was not statistically significant. We cannot comment on potential impact of many variables (e.g. genital hygiene, douching, sexual history) that may impact HPV and vaginal microbiome.**

Logistics: The conclusion emphasizes the potential for these methods in "resource-limited settings." However, the methods describe a cold chain (2-8^{\circ}C transport, -20^{\circ}C storage). In the Discussion, could the authors comment on the stability of samples in the UCM buffer at ambient temperature? This is crucial for true utility in remote areas without cold chain infrastructure.

**Response: We did not include details about the cold chain, but this was achieved in remote areas with the use of cold packs. The manufacturer of the Colli-Pee device with preservative does indicate samples are stable at ambient temperature up to 7 days, however, as ambient temperatures could differ significantly between sites, we opted to provide consistency through the use of ice packs. Further work would be necessary to verify the time of stability of samples kept at ambient temperatures.**

Conclusion:
The study is technically sound and provides important data for HPV surveillance strategies in South Asia. I recommend publication after these minor points are addressed.

Reviewer #6:

Introduction

* Your final aim statement says “sexually active girls and women aged 15–25 years” (line 85), but your Methods section indicates 18–25 in Pakistan and Nepal and “ever-married ≤25” in Bangladesh. Ensure the aim statement aligns with the enrolled age ranges ("18–25 years” overall, or “≤25 years"), maybe with a brief note about country-specific eligibility.

**Response: In Bangladesh early age at marriage is a common practice, so married girls age 15 and older were included. The overall aim statement was intended to cover all three countries, with country-specific inclusion information provided in the methods.**

* You have said that “Pakistan has not yet introduced the HPV vaccine” (lines 54–55). That is no longer accurate: Pakistan launched its first HPV vaccine rollout/campaign beginning September 15, 2025, with plans to integrate it into routine immunization thereafter.

**Response: Thank you, we have added this good news to the introduction (page 5, lines 56-57).**

* The reader will want a tighter argument for why concordance between SCVS and FVU matters in your specific context. I recommend adding 1–2 sentences on whether your primary use case is population-based surveillance, screening uptake, or post-vaccine monitoring, and why urine might be preferable in South Asia (acceptability, cultural norms, logistics, clinic capacity).

**Response: We have revised the introduction and methods to clarify that this comparison was performed in the context of the GLOBE-HPV study, an epidemiologic study of HPV in multiple countries. The introduction states that urine sampling may be preferred over cervical or vaginal sampling in some cultures (page 6, lines 83-85).**

* The introduction can be precise. Consider trimming 1–2 sentences on virology/classification, and use that space to sharpen the study rationale.
**Response: The introduction has been sharpened and rationale clarified.**

Methodology and Results

* The target populations and recruitment strategies vary (Bangladesh: “ever-married ≤25” with stratified systematic sampling; Pakistan: married 18–25; Nepal: sexually active 18–25). This affects both prevalence estimates and potentially concordance if underlying risk factors differ. Please provide baseline participant characteristics by country (age distribution, marital status, parity, education, contraceptive use, recent sexual activity, etc.) and discuss how these differences may influence outcomes.

**Response: The data on participant characteristics and behaviors and their impact on HPV infection will be presented in subsequent GLOBE-HPV publications. The purpose of this analysis was to simply evaluate comparability of HPV detection by two sampling methods. As the concordance was high and similar across multiple settings, the information provides assurance that differences in epidemiologic characteristics and behaviors in these populations was not significant.**

* Self-collection procedures require a little more detail, such as the order of collection and assistance/observation, FVU timing....

**Response: We have added the instructions for sample collection given to participants to the supplemental materials. The order of sample collection was not specified, and this information is added to the methods (page 10, lines 166-167).**

* The high overall agreement/negative agreement (Table 4) likely reflects low prevalence and a large proportion of negative pairs (as your negative agreement is >99%). This is expected but can overstate “equivalence.” Mention this issue in the discussion or emphasize positive concordance and directional discordance.

**Response: It is correct that the negative agreement in low prevalence settings contributes to the high observed concordance. The data in Table 4 also show the lower positive concordance. This is a commonly used approach to compare qualitative (i.e. positive/negative) results between two methods. A statement has been added to the discussion to note this --** It should be noted that in settings with higher HPV prevalence, the positive agreement rates could be expected to be lower, affecting the overall agreement. **(page 18, lines 309-310)**

* Table 3 text: “full discordance ranged from 8.4% (38 HPV types)” appears to be a typo; it should likely be 28 HPV types.

**Response: Yes, the text should read “full discordance ranged from 8.4% (28 types) to 0.7% (HPV 16/18)” but we corrected the typo (page 14, lines 248).**

Reviewer #8

The inclusion criteria and clinical background of the study population are not clearly defined. It is not fully explained whether participants were attending for routine screening, symptomatic evaluation, abnormal cytology follow-up, or other clinical reasons. Because the sample is hospital-based, this may introduce selection bias and limit generalizability to the broader population. The authors should clearly describe the recruitment setting and clinical indications for participation.
**Response: The study setting and rationale has been clarified in the introduction and study description. This analysis was conducted as part of the GLOBE-HPV study. There was not clinical indication for participation. Recruitment was based on goals of the epidemiologic study.**

The cross-sectional nature of the study limits causal interpretation. Some parts of the discussion imply transmission directionality or risk relationships between cervical and anal HPV infection. However, cross-sectional data cannot establish temporality or causation. The manuscript should revise the language to focus strictly on associations rather than inferred transmission pathways.
**Response: We could not identify language in the discussion that commented on transmission or risk relationships between cervical and anal infection. We agree that this should not be there.**

Potential confounding variables are not adequately addressed. Important factors such as sexual behavior, number of sexual partners, smoking status, contraceptive use, immunosuppression, and HIV status could influence HPV prevalence. Without adjustment for these variables, observed associations may be partially confounded. If such data were not collected, this should be clearly acknowledged as a limitation.
**Response: The study was not designed to address the impact of the factors noted (sexual behavior, number of sexual partners, smoking status, contraceptive use, immunosuppression, and HIV status) on HPV prevalence. It was designed to provide evidence that both sampling methods would provide comparable data on HPV detection to allow these factors to be addressed in epidemiologic studies.**

The manuscript does not clearly report whether any samples were excluded due to insufficient DNA, invalid results, or missing data. A participant flow diagram summarizing eligible, excluded, and analyzed cases would improve transparency and align with reporting standards.

**Response: There were no inadequate samples in this study. This has been added to the results (page 13, lines 229).**

Reviewer #9

General Comments:

This study compares self-collected vaginal swabs with first-void urine samples for HPV detection among sexually active girls and women in three South Asian countries. The question is both practical and relevant, particularly in settings where expanding access to cervical cancer screening remains a challenge. Overall, the study appears thoughtfully designed, and the main findings are generally supported by the data presented. A few areas, however, would benefit from additional clarification to improve transparency and reporting.

Major Comments

1. Study Design and Recruitment
It would be helpful to provide more detail about how participants were recruited (e.g., consecutive vs convenience sampling) and to clarify the inclusion and exclusion criteria more explicitly. Please also indicate whether HPV vaccination status was recorded and considered in the analysis. In addition, clarifying whether the order of sample collection (swab vs urine) was standardized would help readers assess potential procedural effects.
**Response: We have clarified that participants were recruited to meet the goals of the GLOBE-HPV. Participant recruitment and explicit inclusion and exclusion criteria are provided (page 9, line 148 – 160).**

**HPV vaccination was recorded as part of the GLOBE-HPV study, but not considered in this analysis, as the objective was simply to evaluate comparability of HPV detection by two sampling methods. In addition, those enrolled in this study were unlikely to be vaccinated as the study was conducted before HPV vaccine rollout in Bangladesh and Nepal and those enrolled in Pakistan were older than those included in the HPV vaccine rollout. The order of sample collection was not specified, and this information is added to the methods (page 9, lines 166 – 167).**

2. Laboratory Procedures
Additional detail regarding the HPV assay would improve reproducibility, including its validation status, limit of detection, and any internal quality controls used. Please also clarify whether laboratory personnel were blinded to specimen type and whether quality assurance procedures were consistent across the participating countries.
**Response: The Seegene HPV assay is a commercial assay that has performed well in interlaboratory HPV proficiency testing (PMID: 39439211 DOI: 10.1002/jmv.70022). The manufacturer does not provide a lower limit of detection. The assay includes internal quality controls for detection of each probe as well as for detection of human DNA. All laboratory methods and quality control measures were harmonized prior to initiating testing and were continuously monitored throughout the study. The sample type could not be blinded as the collection vials were clearly different.**

3.Statistical Methods
The statistical analysis would benefit from clearer reporting. Please specify whether a sample size or power calculation was performed. Confidence intervals should be consistently reported for measures such as sensitivity, specificity, and agreement statistics. If not already included, stratified analyses by country or age group could help explore potential variability across sites.
**Response: The target sample size was set at 247 participants per country, accounting for an anticipated dropout rate of approximately 1%, resulting in an initial enrollment target of 250 participants per country. Assuming a sensitivity of ≥80%, specificity of ≥95%, and an HPV prevalence of approximately 8%, the expected overall agreement would exceed 90%. Under these assumptions, the anticipated Cohen’s kappa would be ≥0.61, indicating substantial agreement according to Landis and Koch (1977). Consequently, a sample size of 250 participants per country was considered sufficient to evaluate agreement between SCVS and FVU. This information was added to methods (page 8, line 133-137).**

**The pattern of sample type concordance did not show statistically significant differences between countries (information added to results section (page 9, line 246 – 247) so country-specific concordance results are not presented. Given the limited age distribution, analysis by age was not considered.**

4.Interpretation of Findings
The conclusions are generally supported by the results; however, where equivalence between sample types is implied, the language may need to be slightly moderated unless a predefined non-inferiority framework was applied. Expanding briefly on possible reasons for discordant results between specimen types would also strengthen the discussion.
**Response: No pre-defined non-inferiority framework was developed. It is recognized that repeat sampling, even with the same method, or repeat testing on the same extract or fresh extract of the original sample has some inherent variation. Since HPV is cell-associated, detection requires including the infected cells, and variations in the ability of repeat sampling to include these cells as well as variations in the number of infected cells and viral copy number contribute to lack of fully discordant results even when same sampling method is used. It is recognized that FVU will have fewer HPV-positive cells than a swab closer to the cervix. Concentration of the FVU sample and use of assays relying on amplification methods can compensate for this. We examined the distribution of viral loads (Ct values) detected in discordant samples for this reason. We do not have further data that could be used to expand the discussion of reasons for discordant results.**

5. Data Availability
The Data Availability Statement indicates that data are available upon reasonable request. Given PLOS ONE’s data-sharing policy, please clarify whether the dataset can be deposited in a public repository or provide a more detailed explanation of any restrictions that prevent open access.
**Response: De-identified individual participant data underlying the results reported in this article will be made available through Vivli (vivli.org), where access requires submission and approval of a formal data access request. This has been, added (page 1, lines 31-33).**

Minor Comments

The manuscript is generally well written and clear, with only minor language refinements needed.
Please ensure consistent terminology (e.g., “first-void urine” throughout).
Tables should clearly specify denominators when percentages are reported.
**Response: We reviewed the manuscript and made a few edits in response to all reviewers. We believe the manuscript readability is improved. Manuscript has been checked for consistent terminology. Denominators have been added to the titles of Tables 3 and 4 (page 14, lines 250; page 15, line 261).**

Overall, this is an important and relevant study. With the clarifications above, the manuscript would be strengthened and better aligned with reporting standards.

Reviewer #11

General Comments:

- This manuscript reports original, multicountry research comparing self-collected vaginal swabs (SCVS) and first-void urine (FVU) for HPV detection among young women in South Asia. The study addresses an important public health question in low- and middle-income countries and contributes valuable evidence for HPV surveillance and screening strategies.

- Minor language editing could further enhance readability.

**Response: We reviewed the manuscript and made a few edits in response to all reviewers. We believe the manuscript readability is improved.**

Abstract

- The abstract is well structured and clearly summarizes the background, objectives, methods, key findings, and conclusions. The study design, sample size, laboratory methods, and main statistical approaches are appropriately described. The results are presented quantitatively and support the stated conclusions. The abstract accurately reflects the content of the manuscript and meets PLOS ONE standards for clarity and completeness

Introduction

- Provides a comprehensive overview of HPV epidemiology, cervical cancer burden, and screening challenges in South Asia. The authors effectively contextualize the need for self-sampling approaches and cite relevant international and regional literature. The rationale for comparing SCVS and FVU is well justified, and the research objectives are clearly stated.

- Lines 44–60 in the Introduction contain detailed country-specific descriptions of cervical cancer burden in Bangladesh, Pakistan, and Nepal. While this information is relevant, this section could be streamlined to improve conciseness and flow. Given that the study focuses on three countries, the authors may consider briefly explaining the rationale for selecting these specific settings (e.g., representativeness, programmatic relevance, epidemiological burden, or participation in the GLOBE-HPV project). In addition, the epidemiological background could be summarized more succinctly by presenting key indicators—such as HPV prevalence, cervical cancer incidence, and mortality rates—for each country, rather than providing extended narrative descriptions. Condensing this section would enhance readability while maintaining its contextual value, and would allow readers to more quickly understand the public health relevance of the selected study sites.

**Response: All the South Asian settings participating in the GLOBE-HPV study were included. Their inclusion was based on the aims of the GLOBE-HPV study. This comparison was performed to provide evidence on the equivalence of HPV detection with first-void urine and self-collected vaginal swabs. The extended narrative was included because data on HPV prevalence, cervical cancer incidence and mortality rates are not clear for each country. We have tried to shorten this by combining descriptions into one paragraph.**

Materials and Methods

The manuscript states that in Bangladesh and Pakistan, participants were required to be married, whereas in Nepal eligibility was based on being sexually active regardless of marital status.

Could the authors please clarify the rationale for this difference in inclusion criteria across countries?

Specifically:

- Was marital status used as a proxy for sexual activity in Bangladesh and Pakistan due to sociocultural or ethical considerations?

**Response:** **Yes, marital status was used as a proxy for sexual activity in Bangladesh and Pakistan due to sociocultural, religious, or ethical considerations. Asking about sexual activity outside of marriage is culturally sensitive and could cause repercussions for the participants. This explanation has been added (page 9, lines 156-157).**

- Were there regulatory or institutional constraints that prevented recruitment of unmarried sexually active women in those settings?

**Response:** **As noted above, in Bangladesh and Pakistan this approach is culturally inappropriate.**

- How might this difference affect comparability of HPV prevalence and concordance estimates across the three countries?

**Response: We do not anticipate that will affect comparability. The cultures of Bangladesh and Pakistan result in near concordance between sexually active women and married women.**

A brief explanation in the Methods section, and possibly a note in the Limitations section addressing potential selection bias or differences in sexual behavior patterns, would strengthen transparency and interpretation of cross-country comparisons.

**Response:** **As cross-country comparisons is not the intent of this manuscript, and we don’t include this in our conclusions, we modified the limitations to clarify the study findings may not be applicable to all populations.**

Sample Size

Could the authors please clarify:

- Whether a formal sample size or power calculation was performed for this concordance analysis?

- If so, which assumptions were used (expected HPV prevalence, anticipated agreement/Kappa value, precision, alpha level)?

- Whether the target of approximately 250 participants per country was based on logistical considerations, cohort design, or statistical requirements of the GLOBE-HPV project?

**Response:** **The target sample size was set at 247 participants per country, accounting for an anticipated dropout rate of approximately 1%, resulting in an initial enrollment target of 250 participants per country. Assuming a sensitivity of ≥80%, specificity of ≥95%, and an HPV prevalence of approximately 8%, the expected overall agreement would exceed 90%. Under these assumptions, the anticipated Cohen’s kappa would be ≥0.61, indicating substantial agreement according to Landis and Koch (1977). Consequently, a sample size of 250 participants per country was considered sufficient to evaluate agreement between SCVS and FVU. This information was added to method section (page 8, lines 133 – 137).**

Results

- The manuscript reports no inadequate test results; however, additional clarification on data completeness (e.g., missing demographic or behavioral variables) would improve transparency.

- The description of discordant cases could be expanded, for example by indicating whether discordance was more frequent in specific subgroups (country, age range, or HPV type categories).

**Response: As the focus of this study is comparison of two sampling methods, the meta-data on demographic and behavioral variables is not included. These will be discussed in GLOBE-HPV publications focusing on these issues. However, overall, there is little missing data and no significant differences between sites.**

**We feel the message of the paper is the similarity of results across countries. The limited number of discordant samples precludes an analysis of individual types and ages. The impact of sampling on type distribution can be seen in Table 2.**

Discussion

The Discussion section is generally well written and provides an appropriate interpretation of the study findings in relation to existing literature. The authors successfully contextualize their results within prior studies on self-sampling for HPV detection and highlight the relevance for surveillance in low- and middle-income settings.

However, several aspects could be strengthened:

**- Balance between interpretation and speculation** Some explanations regarding differences in sensitivity and concordance between SCVS and FVU appear speculative and would benefit from stronger empirical support or clearer citation of supporting evidence.

**Response: We are not clear which explanations appear speculative. We have reviewed this discussion to clarify**

**- Cross-country comparability** Given the differences in inclusion criteria and recruitment strategies across countries, the authors should more explicitly discuss how these variations may influence the comparability of findings and interpretation of pooled analyses.

**Response: Differences in target populations were a component of the GLOBE-HPV study. Pooled analyses provide sufficient numbers for comparison of the two sample types, with the knowledge that these are collected in different populations. We conducted an analysis verifying there was no statistical significance in concordance between sites prior to pooling the data. Only the full GLOBE-HPV data sets will be used for comparison across countries.**

**- Public health and implementation implications** While the potential use of SCVS and FVU in surveillance programs is mentioned, further discussion on operational feasibility, cost considerations, and integration into existing screening systems in South Asia would enhance the practical relevance of the findings.

**Response: We did not conduct an analysis of operational feasibility or cost as these differ greatly between countries based on local suppliers and tariffs. Further, while there is interest in self-sampling methods for screening, this study did not address screening. Our study findings could be used to allow countries to select sampling methods that fit their specific conditions for studies validating their results for screening, based on cervical disease endpoints.**

**- Limitations and potential biases** The limitations section could be expanded to more clearly address potential selection bias, reporting bias related to self-sampling, and variability in laboratory processing across sites.

**Response: As the study included all women in the GLOBE-HPV study eligible for evaluation, there no selection bias, the refusal rate was very low, so bias related to reluctance for self-sampling is not a concern. Further, the use of harmonized standard operating procedures was a hallmark of this study, and variations in laboratory processing across sites were kept to a minimum. The most important limitation is that use of other sampling devices, media, extraction and assays could affect extent of agreement between sampling methods. We have revised the limitations sections to highlight this and included the possibility of population effects and site differences. We have also added the limitation that neither sample was compared to clinician collected samples.**

**Below we respond to the general comments we received as MS-Word file attachment:**

**General Comments:**

- This manuscript reports original, multicountry research comparing self-collected vaginal swabs (SCVS) and first-void urine (FVU) for HPV detection among young women in South Asia. The study addresses an important public health question in low- and middle-income countries and contributes valuable evidence for HPV surveillance and screening strategies.

- Minor language editing could further enhance readability.

**Response: We reviewed the manuscript and made a few edits in response to both reviewers. We believe the manuscript readability is improved.**

Abstract

- The abstract is well structured and clearly summarizes the background, objectives, methods, key findings, and conclusions. The study design, sample size, laboratory methods, and main statistical approaches are appropriately described. The results are presented quantitatively and support the stated conclusions. The abstract accurately reflects the content of the manuscript and meets PLOS ONE standards for clarity and completeness

Introduction

- Provides a comprehensive overview of HPV epidemiology, cervical cancer burden, and screening challenges in South Asia. The authors effectively contextualize the need for self-sampling approaches and cite relevant international and regional literature. The rationale for comparing SCVS and FVU is well justified, and the research objectives are clearly stated.

- Lines 44–60 in the Introduction contain detailed country-specific descriptions of cervical cancer burden in Bangladesh, Pakistan, and Nepal. While this information is relevant, this section could be streamlined to improve conciseness and flow. Given that the study focuses on three countries, the authors may consider briefly explaining the rationale for selecting these specific settings (e.g., representativeness, programmatic relevance, epidemiological burden, or participation in the GLOBE-HPV project). In addition, the epidemiological background could be summarized more succinctly by presenting key indicators—such as HPV prevalence, cervical cancer incidence, and mortality rates—for each country, rather than providing extended narrative descriptions. Condensing this section would enhance readability while maintaining its contextual value, and would allow readers to more quickly understand the public health relevance of the selected study sites.

**Response: All the South Asian settings participating in the GLOBE-HPV study were included. Their inclusion was based on the aims of the GLOBE-HPV study. This comparison was performed to provide evidence on the equivalence of HPV detection with first-void urine and self-collected vaginal swabs. The extended narrative was included because data on HPV prevalence, cervical cancer incidence and mortality rates are not clear for each country. We have tried to shorten this by combining descriptions into one paragraph.**

Materials and Methods

The manuscript states that in Bangladesh and Pakistan, participants were required to be married, whereas in Nepal eligibility was based on being sexually active regardless of marital status.

**Response: Provided at the end of each question**

Could the authors please clarify the rationale for this difference in inclusion criteria across countries?

Specifically:

- Was marital status used as a proxy for sexual activity in Bangladesh and Pakistan due to sociocultural or ethical considerations? **Yes, marital status was used as a proxy for sexual activity in Bangladesh and Pakistan due to sociocultural, religious, or ethical considerations. Asking about sexual activity outside of marriage is culturally sensitive and could cause repercussions for the participants. This explanation has been added.**

- Were there regulatory or institutional constraints that prevented recruitment of unmarried sexually active women in those settings? **As noted above, in Bangladesh and Pakistan this approach is culturally inappropriate.**

- How might this difference affect comparability of HPV prevalence and concordance estimates across the three countries? **We do not anticipate that will affect comparability. The cultures of Bangladesh and Pakistan result in near concordance between sexually active women and married women.**

A brief explanation in the Methods section, and possibly a note in the Limitations section addressing potential selection bias or differences in sexual behavior patterns, would strengthen transparency and interpretation of cross-country comparisons. **As cross-country comparisons is not the intent of this manuscript, and we don’t include this in our conclusions, we modified the limitations to clarify the study findings may not be applicable to all populations.**

Sample Size

Could the authors please clarify:

- Whether a formal sample size or power calculation was performed for this concordance analysis? Yes

- If so, which assumptions were used (expected HPV prevalence, anticipated agreement/Kappa value, precision, alpha level)? **Response:** **The target sample size was set at 247 participants per country, accounting for an anticipated dropout rate of approximately 1%, resulting in an initial enrollment target of 250 participants per country. Assuming a sensitivity of ≥80%, specificity of ≥95%, and an HPV prevalence of approximately 8%, the expected overall agreement would exceed 90%. Under these assumptions, the anticipated Cohen’s kappa would be ≥0.61, indicating substantial agreement according to Landis and Koch (1977). Consequently, a sample size of 250 participants per country was considered sufficient to evaluate agreement between SCVS and FVU. This information was added to methods**

- Whether the target of approximately 250 participants per country was based on logistical considerations, cohort design, or statistical requirements of the GLOBE-HPV project? **The target of 250 participants per country was based on statistical requirements of the GLOBE-HPV project.**

Results

- The manuscript reports no inadequate test results; however, additional clarification on data completeness (e.g., missing demographic or behavioral variables) would improve transparency.

- The description of discordant cases could be expanded, for example by indicating whether discordance was more frequent in specific subgroups (country, age range, or HPV type categories).

**Response: As the focus of this study is comparison of two sampling methods, the meta-data on demographic and behavioral variables is not included. These will be discussed in GLOBE-HPV publications focusing on these issues. However, overall, there is little missing data and no significant differences between sites.**

**We feel the message of the paper is the similarity of results across countries. The limited number of discordant samples precludes an analysis of individual types and ages. The impact of sampling on type distribution can be seen in Table 2.**

Discussion

The Discussion section is generally well written and provides an appropriate interpretation of the study findings in relation to existing literature. The authors successfully contextualize their results within prior studies on self-sampling for HPV detection and highlight the relevance for surveillance in low- and middle-income settings.

However, several aspects could be strengthened:

**- Balance between interpretation and speculation** Some explanations regarding differences in sensitivity and concordance between SCVS and FVU appear speculative and would benefit from stronger empirical support or clearer citation of supporting evidence.

**Response: We are not clear which explanations appear speculative. We have reviewed this discussion to clarify**

**- Cross-country comparability** Given the differences in inclusion criteria and recruitment strategies across countries, the authors should more explicitly discuss how these variations may influence the comparability of findings and interpretation of pooled analyses.

**Response: Differences in target populations were a component of the GLOBE-HPV study. Pooled analyses provide sufficient numbers for comparison of the two sample types, with the knowledge that these are collected in different populations. We conducted an analysis verifying there was no statistical significance in concordance between sites prior to pooling the data. Only the full GLOBE-HPV data sets will be used for comparison across countries.**

**- Public health and implementation implications** While the potential use of SCVS and FVU in surveillance programs is mentioned, further discussion on operational feasibility, cost considerations, and integration into existing screening systems in South Asia would enhance the practical relevance of the findings.

**We did not conduct an analysis of operational feasibility or cost as these differ greatly between countries based on local suppliers and tariffs. Further, while there is interest in self-sampling methods for screening, this study did not address screening. Our study findings could be used to allow countries to select sampling methods that fit their specific conditions for studies validating their results for screening, based on cervical disease endpoints.**

**- Limitations and potential biases** The limitations section could be expanded to more clearly address potential selection bias, reporting bias related to self-sampling, and variability in laboratory processing across sites.

**As the study included all women in the GLOBE-HPV study eligible for evaluation, there is not selection bias, the refusal rate was very low, so bias related to reluctance for self-sampling is not a concern. Further, the use of harmonized standard operating procedures was a hallmark of this study, and variations in laboratory processing across sites was kept to a minimum. The most important limitation is that use of other sampling devices, media, extraction and assays could affect extent of agreement between sampling methods. We have revised the limitations sections to highlight this and included the possibility of population effects and site differences. We have also added the limitation that neither sample was compared to clinician collected samples.**

**Comments PLoS One:**

The article entitled, “Comparison of self-collected vaginal swabs and first-void urine for detection of human papillomavirus in sexually active girls and women in three South Asian countries,” by Telele et al., describes a cross-sectional study wherein the authors compared two non-invasive sampling methods for HPV DNA detection. The authors collected 753 samples from sexually active females ages 15–25 in Bangladesh, Pakistan, and Nepal using two methods, i.e., self-collected vaginal swabs (SCVS) and first-void urine (FVU). The authors used a harmonized protocol not only to assess HPV prevalence but also to determine concordance between the two sample types.

Though the study appears to be scientifically sound, and the manuscript is well organized and prepared, there are few changes required to strengthen the objectives and outcomes of the study. The manuscript can be accepted for publication in the PLoS One after replies to the following comments are addressed satisfactorily:

1.	The authors cited similar studies that have compared self-collected vs urine samples for HPV. The authors should mention with clarity what is the uniqueness/novelty of the present study.

**Response: While self-collected swabs and urine samples have been studied as methods for HPV detection, the methods used, including collection devices, preservatives, processing, extraction and assays all differ. Our results add to options that have been evaluated and include the ability to perform the collection and testing in a harmonized manner. Further, we included an exploration of the impact of viral load on the concordance between samples. We modified the introduction to clarify this.**

2.	In the introduction section, instead of over emphasizing the HPV background and self-sampling the authors should focus on the research question and what knowledge gap is being filled by carrying out the present study.

**Response: The introduction has been extensively modified to emphasize the knowledge gap being addressed.**

3.	The authors should provide the justification of cohort chosen especially the reason for age of 15-25 years and if / how it is related to HPV vaccination. Why do authors have not included broader age range? The reasoning behind the narrative should be provided in the manuscript as it would strengthen the manuscript.

**Response: The cohorts were formed based on the aims of the GLOBE-HPV study. The age groups for the comparison of sampling methods was selected to primarily because of the likelihood of HPV acquisition. In addition, this age group is generally considered for “catch-up” vaccination, so providing an indication of exposure to vaccine types could be of importance to countries planning HPV vaccine campaigns.**

4.	The recruitment strategies detailed in the present study differed by country. Such as, the authors carried clinical based sampling in Nepal; demographic surveillance in Pakistan and sampling of married women in Bangladesh. The impact of recruitment strategy can be on the results / observations of the study. The authors should provide a clarification on the approach.

**Response: Marital status was used as a proxy for sexual activity in Bangladesh and Pakistan due to sociocultural, religious, or ethical considerations. Asking about sexual activity outside of marriage is culturally sensitive and could cause repercussions for the participants. This explanation has been added. We do not think that this difference in recruitment impacts study findings as the cultures of Bangladesh and Pakistan result in near concordance between sexually active women and married women.**

5.	In discussion section, the authors could provide information on comparing the practical advantages between the two methods of sampling. They should also discuss how the present study outcomes can have influence on screening strategies. The authors can summarize the major findings/takeaways of the present study in conclusion.

**Response: The major takeaway of the present study is included in the conclusion, that is, both sampling methods give very similar HR-HPV results. We did not collect data on practical advantages between the two methods. These would include preference of the women (not addressed), costs of materials and disposal (different in each setting due to local importing suppliers and tariffs), and laboratory facilities (standardized and key assay instrument provided as part of GLOBE-HPV).**

6.	As the samples were self-collected, the authors should acknowledge the limitations of the method because of absence of clinician / trained personnel collected samples as it can impact the outcome of the result. The authors should mention other limitations too.

**Response: The biggest limitation to the findings is that they apply to the specific sample collection tools, media, processing, extraction and assays used in this study and may not be generalizable to all methods. Lack of clinician collected samples as comparator was added to the limitations and the potential impact of population-specific factors and laboratory variations was added.**

7.	The authors should provide the probable reasons for the observations that why Nepal had higher HPV prevalence than other two countries. Further, authors should mention if they have any plans for future studies to follow up or some other objectives.

**Response: This study is focused on comparability of sampling methods. Discussion of country-specific differences in HPV prevalence as well as persistence will be addressed in the GLOBE-HPV publications.**

8.	The authors should ensure consistency in use of HPV-related terms. Such as at some places for High Risk ‘hr’ is used while at other places ‘HR’ is used. Authors should check the manuscript to make sure about uniformity.

**Response: Checked.**

9.	Authors should make sure that all web references have accessed date and formatted as per PLoS One requirements. Also, make sure that all the references are cited and citations provided meet the PLoS One requirements.

**Response: Checked**
