# Supplementary material for: Comparison of self-collected vaginal swabs and first-void urine for detection of human papillomavirus in sexually active girls and women in three South Asian countries
Source: PLoS One. 2026 Jun 12;21(6):e0350049. doi: 10.1371/journal.pone.0350049 (PMC13262861; doi:10.1371/journal.pone.0350049)
Supplement: Peer review history

# Peer review: Decision Letter 0

**DOI:** 10.1371/journal.pone.0350049.r001

**Authors:** Ramokone Lebelo

23 Mar 2026

PONE-D-25-43925

Comparison of self-collected vaginal swabs and first-void urine for detection of human papillomavirus in sexually active girls and women in three South Asian countries

PLOS One

Dear Dr. Telele,

Thank you for submitting your manuscript to PLOS ONE. After careful consideration, we feel that it has merit but does not fully meet PLOS ONE’s publication criteria as it currently stands. Therefore, we invite you to submit a revised version of the manuscript that addresses the points raised during the review process.

==============================

Thank you for submitting your paper to PLOS One for potential publication.

This is relevant and important study in the era of cervical cancer elimination and WHO 2030 goals.

Please address the comments from Reviewer 1 and 9 fully, having a response for every comment. Please also pay attention to the feedback from reviewers 2, 4, 6, 8, and 11.

These are critical issues to address:

1. The novelty of the study must be made clear by the authors since this is not the first paper to report the comparison of FVU and self collected samples.

2. The study participants have different characteristics, from all three sites, please explain the rationale and how bias would be avoided. This is includes age, marital status etc.

In some instances the first void urine was not collected however this is not accounted for in the analysis and the title, this may compromise the results.

3. How was consistency in sample collection and recruitment ensured throughout the three sites?

==============================

Please submit your revised manuscript by May 04 2026 11:59PM. If you will need more time than this to complete your revisions, please reply to this message or contact the journal office at plosone@plos.org. When you're ready to submit your revision, log on to https://www.editorialmanager.com/pone/ and select the 'Submissions Needing Revision' folder to locate your manuscript file.

Please include the following items when submitting your revised manuscript:

- A letter that responds to each point raised by the academic editor and reviewer(s). You should upload this letter as a separate file labeled 'Response to Reviewers'.
- A marked-up copy of your manuscript that highlights changes made to the original version. You should upload this as a separate file labeled 'Revised Manuscript with Track Changes'.
- An unmarked version of your revised paper without tracked changes. You should upload this as a separate file labeled 'Manuscript'.

If you would like to make changes to your financial disclosure, please include your updated statement in your cover letter. Guidelines for resubmitting your figure files are available below the reviewer comments at the end of this letter.

If applicable, we recommend that you deposit your laboratory protocols in protocols.io to enhance the reproducibility of your results. Protocols.io assigns your protocol its own identifier (DOI) so that it can be cited independently in the future. For instructions see: https://journals.plos.org/plosone/s/submission-guidelines#loc-laboratory-protocols. Additionally, PLOS ONE offers an option for publishing peer-reviewed Lab Protocol articles, which describe protocols hosted on protocols.io. Read more information on sharing protocols at https://plos.org/protocols?utm_medium=editorial-email&utm_source=authorletters&utm_campaign=protocols.

We look forward to receiving your revised manuscript.

Kind regards,

Ramokone Lisbeth Lebelo, Ph.D

Academic Editor

PLOS One

Journal Requirements:

When submitting your revision, we need you to address these additional requirements.

1. Please ensure that your manuscript meets PLOS ONE's style requirements, including those for file naming. The PLOS ONE style templates can be found at

https://journals.plos.org/plosone/s/file?id=wjVg/PLOSOne_formatting_sample_main_body.pdf and https://journals.plos.org/plosone/s/file?id=ba62/PLOSOne_formatting_sample_title_authors_affiliations.pdf

2. Thank you for stating in your Funding Statement:

[This research was supported by Gates Foundation under Investment ID INV-046024 and Swedish Government through IVI Europe Regional Office (IERO)].

Please provide an amended statement that declares *all* the funding or sources of support (whether external or internal to your organization) received during this study, as detailed online in our guide for authors at http://journals.plos.org/plosone/s/submit-now. Please also include the statement “There was no additional external funding received for this study.” in your updated Funding Statement.

Please include your amended Funding Statement within your cover letter. We will change the online submission form on your behalf.

3. Please note that funding information should not appear in any section or other areas of your manuscript. We will only publish funding information present in the Funding Statement section of the online submission form. Please remove any funding-related text from the manuscript.

4. We note that you have indicated that there are restrictions to data sharing for this study. For studies involving human research participant data or other sensitive data, we encourage authors to share de-identified or anonymized data. However, when data cannot be publicly shared for ethical reasons, we allow authors to make their data sets available upon request. For information on unacceptable data access restrictions, please see http://journals.plos.org/plosone/s/data-availability#loc-unacceptable-data-access-restrictions.

Before we proceed with your manuscript, please address the following prompts:

a) If there are ethical or legal restrictions on sharing a de-identified data set, please explain them in detail (e.g., data contain potentially identifying or sensitive patient information, data are owned by a third-party organization, etc.) and who has imposed them (e.g., a Research Ethics Committee or Institutional Review Board, etc.). Please also provide contact information for a data access committee, ethics committee, or other institutional body to which data requests may be sent.

b) If there are no restrictions, please upload the minimal anonymized data set necessary to replicate your study findings to a stable, public repository and provide us with the relevant URLs, DOIs, or accession numbers. Please see http://www.bmj.com/content/340/bmj.c181.long for guidelines on how to de-identify and prepare clinical data for publication. For a list of recommended repositories, please see https://journals.plos.org/plosone/s/recommended-repositories. You also have the option of uploading the data as Supporting Information files, but we would recommend depositing data directly to a data repository if possible.

Please update your Data Availability statement in the submission form accordingly.

5. In the online submission form, you indicated that [De-identified data can be made available upon reasonable request.].

All PLOS journals now require all data underlying the findings described in their manuscript to be freely available to other researchers, either 1. In a public repository, 2. Within the manuscript itself, or 3. Uploaded as supplementary information.

This policy applies to all data except where public deposition would breach compliance with the protocol approved by your research ethics board. If your data cannot be made publicly available for ethical or legal reasons (e.g., public availability would compromise patient privacy), please explain your reasons on resubmission and your exemption request will be escalated for approval.

If the reviewer comments include a recommendation to cite specific previously published works, please review and evaluate these publications to determine whether they are relevant and should be cited. There is no requirement to cite these works unless the editor has indicated otherwise.

Additional Editor Comments:

Thank you for submitting your paper to PLOS One for review.

We sincerely apologize for the prolonged period of review, it is not the standard of PLOS One.

The decision on your paper was based on the reviewer's comments as major comments.

Please address the comments and reviews of Reviewer 1 and Reviewer 9 fully.

[Note: HTML markup is below. Please do not edit.]

Reviewers' comments:

Reviewer's Responses to Questions

**Comments to the Author**

1. Is the manuscript technically sound, and do the data support the conclusions?

The manuscript must describe a technically sound piece of scientific research with data that supports the conclusions. Experiments must have been conducted rigorously, with appropriate controls, replication, and sample sizes. The conclusions must be drawn appropriately based on the data presented.

Reviewer #1: Partly

Reviewer #2: Yes

Reviewer #3: Yes

Reviewer #4: Yes

Reviewer #5: Yes

Reviewer #6: Yes

Reviewer #7: Yes

Reviewer #8: Yes

Reviewer #9: Yes

Reviewer #10: Yes

Reviewer #11: Yes

**********

2. Has the statistical analysis been performed appropriately and rigorously?

Reviewer #1: Yes

Reviewer #2: Yes

Reviewer #3: Yes

Reviewer #4: Yes

Reviewer #5: Yes

Reviewer #6: Yes

Reviewer #7: Yes

Reviewer #8: Yes

Reviewer #9: Yes

Reviewer #10: Yes

Reviewer #11: Yes

**********

3. Have the authors made all data underlying the findings in their manuscript fully available?

The PLOS Data policy requires authors to make all data underlying the findings described in their manuscript fully available without restriction, with rare exception (please refer to the Data Availability Statement in the manuscript PDF file). The data should be provided as part of the manuscript or its supporting information, or deposited to a public repository. For example, in addition to summary statistics, the data points behind means, medians and variance measures should be available. If there are restrictions on publicly sharing data—e.g. participant privacy or use of data from a third party—those must be specified.

Reviewer #1: Yes

Reviewer #2: Yes

Reviewer #3: Yes

Reviewer #4: No

Reviewer #5: Yes

Reviewer #6: Yes

Reviewer #7: Yes

Reviewer #8: Yes

Reviewer #9: No

Reviewer #10: Yes

Reviewer #11: Yes

**********

4. Is the manuscript presented in an intelligible fashion and written in standard English?

PLOS ONE does not copyedit accepted manuscripts, so the language in submitted articles must be clear, correct, and unambiguous. Any typographical or grammatical errors should be corrected at revision, so please note any specific errors here.

Reviewer #1: Yes

Reviewer #2: Yes

Reviewer #3: Yes

Reviewer #4: Yes

Reviewer #5: Yes

Reviewer #6: Yes

Reviewer #7: Yes

Reviewer #8: Yes

Reviewer #9: Yes

Reviewer #10: Yes

Reviewer #11: No

**********

5. Review Comments to the Author

Please use the space provided to explain your answers to the questions above. You may also include additional comments for the author, including concerns about dual publication, research ethics, or publication ethics. (Please upload your review as an attachment if it exceeds 20,000 characters)

Reviewer #1: The article entitled, “Comparison of self-collected vaginal swabs and first-void urine for detection of human papillomavirus in sexually active girls and women in three South Asian countries,” by Telele et al., describes a cross-sectional study wherein the authors compared two non-invasive sampling methods for HPV DNA detection. The authors collected 753 samples from sexually active females ages 15–25 in Bangladesh, Pakistan, and Nepal using two methods, i.e., self-collected vaginal swabs (SCVS) and first-void urine (FVU). The authors used a harmonized protocol not only to assess HPV prevalence but also to determine concordance between the two sample types.

Though the study appears to be scientifically sound, and the manuscript is well organized and prepared, there are few changes required to strengthen the objectives and outcomes of the study. The manuscript can be accepted for publication in the PLoS One after replies to the following comments are addressed satisfactorily:

1. The authors cited similar studies that have compared self-collected vs urine samples for HPV. The authors should mention with clarity what is the uniqueness/novelty of the present study.

2. In the introduction section, instead of over emphasizing the HPV background and self-sampling the authors should focus on the research question and what knowledge gap is being filled by carrying out the present study.

3. The authors should provide the justification of cohort chosen especially the reason for age of 15-25 years and if / how it is related to HPV vaccination. Why do authors have not included broader age range? The reasoning behind the narrative should be provided in the manuscript as it would strengthen the manuscript.

4. The recruitment strategies detailed in the present study differed by country. Such as, the authors carried clinical based sampling in Nepal; demographic surveillance in Pakistan and sampling of married women in Bangladesh. The impact of recruitment strategy can be on the results / observations of the study. The authors should provide a clarification on the approach.

5. In discussion section, the authors could provide information on comparing the practical advantages between the two methods of sampling. They should also discuss how the present study outcomes can have influence on screening strategies. The authors can summarize the major findings/takeaways of the present study in conclusion.

6. As the samples were self-collected, the authors should acknowledge the limitations of the method because of absence of clinician / trained personnel collected samples as it can impact the outcome of the result. The authors should mention other limitations too.

7. The authors should provide the probable reasons for the observations that why Nepal had higher HPV prevalence than other two countries. Further, authors should mention if they have any plans for future studies to follow up or some other objectives.

8. The authors should ensure consistency in use of HPV-related terms. Such as at some places for High Risk ‘hr’ is used while at other places ‘HR’ is used. Authors should check the manuscript to make sure about uniformity.

9. Authors should make sure that all web references have accessed date and formatted as per PLoS One requirements. Also, make sure that all the references are cited and citations provided meet the PLoS One requirements.

Reviewer #2: This is an automated report for PONE-D-25-43925. This report was solicited by the PLOS One editorial team and provided by ScreenIT.

ScreenIT is an independent group of scientists developing automated tools that analyze academic papers. A set of automated tools screened your submitted manuscript and provided the report below. Each tool was created by your academic colleagues with the goal of helping authors. The tools look for factors that are important for transparency, rigor and reproducibility, and we hope that the report might help you to improve reporting in your manuscript. Within the report you will find links to more information about the items that the tools check. These links include helpful papers, websites, or videos that explain why the item is important. While our screening tools aim to improve and maintain quality standards they may, on occasion, miss nuances specific to your study type or flag something incorrectly. Each tool has limitations that are described on the ScreenIT website. The tools screen the main file for the paper; they are not able to screen supplements stored in separate files. Please note that the Academic Editor had access to these comments while making a decision on your manuscript. The Academic Editor may ask that issues flagged in this report be addressed. If you would like to provide feedback on the ScreenIT tool, please email the team at ScreenIt@bih-charite.de. If you have questions or concerns about the review process, please contact the PLOS One office at plosone@plos.org.

Reviewer #3: Excellent work! This is a very important piece of research regarding Human Papilloma Virus infection in a young sexually active population. The methods used are rigorous and reproducable. They serve as the template for future work on this topic.

Reviewer #4: This manuscript presents a valuable multi-country study evaluating the concordance between self-collected vaginal swabs (SCVS) and first-void urine (FVU) for HPV detection among young women in Bangladesh, Nepal, and Pakistan. The study addresses a critical public health need in resource-limited settings. The methodology is robust, the sample size is adequate, and the manuscript is well-written. The harmonized protocol across three countries is a significant strength.

However, I have a few minor comments and queries that, if addressed, would further strengthen the manuscript.

Specific Comments:

Sample Validity: The results state (Page 12, Line 196) that "No inadequate test results were reported from either sample type." Given the nature of self-collection and the large sample size (N=753 pairs), a 0% invalid rate is exceptionally evident of high-quality supervision or robust DNA extraction methods. Could the authors briefly clarify in the Methods section if any participants were excluded prior to the final N=753 count due to sample insufficiency, or if the "UCM" buffer and extraction protocol truly yielded 100% valid DNA for all collected samples? This detail speaks to the feasibility of this method in real-world surveillance.

Data Availability: The authors have indicated that data is available "upon reasonable request" due to ethical restrictions. Please ensure this complies with PLOS ONE’s strict data availability policy. If the data contains sensitive patient information that prevents public deposition, the specific ethical restrictions and the contact information for a non-author institutional body (e.g., an Ethics Committee) should be clearly stated in the final declaration, rather than just the authors' contact.

Cycle Threshold (Ct) Analysis: The comparison of Ct values between concordant and discordant samples is a very insightful addition (Figure 1). It clearly demonstrates that discordance is driven by low viral loads. It would be beneficial to briefly discuss the clinical implication of this: are the "missed" infections in discordant cases likely to be clinically significant (e.g., persistent infections) or transient low-load infections?

Demographics: The study population varies slightly by country (e.g., married women in Bangladesh/Pakistan vs. sexually active women in Nepal). While the authors acknowledge this in the limitations, a brief sentence in the Discussion on whether marital status (and implied sexual behavior differences) might impact the vaginal microbiome or viral load—and thus detection concordance—would be interesting, though not mandatory.

Logistics: The conclusion emphasizes the potential for these methods in "resource-limited settings." However, the methods describe a cold chain (2-8^{\circ}C transport, -20^{\circ}C storage). In the Discussion, could the authors comment on the stability of samples in the UCM buffer at ambient temperature? This is crucial for true utility in remote areas without cold chain infrastructure.

Conclusion:

The study is technically sound and provides important data for HPV surveillance strategies in South Asia. I recommend publication after these minor points are addressed.

Reviewer #5: This is a very important study for public health in South Asia. Comparing self-collected vaginal swabs and urine for HPV testing is a great idea, especially for reaching women in resource-limited settings.

I really appreciate that you collected data from three different countries (Bangladesh, Nepal, and Pakistan). The results are clear and promising—showing that both methods work well for detecting high-risk HPV. This will be very helpful for future vaccination monitoring programs.

I have no changes to request. Congratulations on a successful multi-country study.

Reviewer #6: Introduction

* Your final aim statement says “sexually active girls and women aged 15–25 years” (line 85), but your Methods section indicates 18–25 in Pakistan and Nepal and “ever-married ≤25” in Bangladesh. Ensure the aim statement aligns with the enrolled age ranges ("18–25 years” overall, or “≤25 years"), maybe with a brief note about country-specific eligibility.

* You have said that “Pakistan has not yet introduced the HPV vaccine” (lines 54–55). That is no longer accurate: Pakistan launched its first HPV vaccine rollout/campaign beginning September 15, 2025, with plans to integrate it into routine immunization thereafter.

* The reader will want a tighter argument for why concordance between SCVS and FVU matters in your specific context. I recommend adding 1–2 sentences on whether your primary use case is population-based surveillance, screening uptake, or post-vaccine monitoring, and why urine might be preferable in South Asia (acceptability, cultural norms, logistics, clinic capacity).

* The introduction can be precise. Consider trimming 1–2 sentences on virology/classification, and use that space to sharpen the study rationale.

Methodology and Results

* The target populations and recruitment strategies vary (Bangladesh: “ever-married ≤25” with stratified systematic sampling; Pakistan: married 18–25; Nepal: sexually active 18–25). This affects both prevalence estimates and potentially concordance if underlying risk factors differ. Please provide baseline participant characteristics by country (age distribution, marital status, parity, education, contraceptive use, recent sexual activity, etc.) and discuss how these differences may influence outcomes.

* Self-collection procedures require a little more detail, such as the order of collection and assistance/observation, FVU timing....

* The high overall agreement/negative agreement (Table 4) likely reflects low prevalence and a large proportion of negative pairs (as your negative agreement is >99%). This is expected but can overstate “equivalence.” Mention this issue in the discussion or emphasize positive concordance and directional discordance.

* Table 3 text: “full discordance ranged from 8.4% (38 HPV types)” appears to be a typo; it should likely be 28 HPV types.

Reviewer #7: Excellent paper and an important topic. This paper discusses an important and emerging topic. It helps inform our current knowledge and will prove beneficial for the medical community. The methodology is rigorous.

Reviewer #8: Thanks for your manuscript.

Comments

The inclusion criteria and clinical background of the study population are not clearly defined. It is not fully explained whether participants were attending for routine screening, symptomatic evaluation, abnormal cytology follow-up, or other clinical reasons. Because the sample is hospital-based, this may introduce selection bias and limit generalizability to the broader population. The authors should clearly describe the recruitment setting and clinical indications for participation.

The cross-sectional nature of the study limits causal interpretation. Some parts of the discussion imply transmission directionality or risk relationships between cervical and anal HPV infection. However, cross-sectional data cannot establish temporality or causation. The manuscript should revise the language to focus strictly on associations rather than inferred transmission pathways.

Potential confounding variables are not adequately addressed. Important factors such as sexual behavior, number of sexual partners, smoking status, contraceptive use, immunosuppression, and HIV status could influence HPV prevalence. Without adjustment for these variables, observed associations may be partially confounded. If such data were not collected, this should be clearly acknowledged as a limitation.

The manuscript does not clearly report whether any samples were excluded due to insufficient DNA, invalid results, or missing data. A participant flow diagram summarizing eligible, excluded, and analyzed cases would improve transparency and align with reporting standards.

Reviewer #9: General comments

This study compares self-collected vaginal swabs with first-void urine samples for HPV detection among sexually active girls and women in three South Asian countries. The question is both practical and relevant, particularly in settings where expanding access to cervical cancer screening remains a challenge. Overall, the study appears thoughtfully designed, and the main findings are generally supported by the data presented. A few areas, however, would benefit from additional clarification to improve transparency and reporting.

Major Comments

1. Study Design and Recruitment

It would be helpful to provide more detail about how participants were recruited (e.g., consecutive vs convenience sampling) and to clarify the inclusion and exclusion criteria more explicitly. Please also indicate whether HPV vaccination status was recorded and considered in the analysis. In addition, clarifying whether the order of sample collection (swab vs urine) was standardized would help readers assess potential procedural effects.

2. Laboratory Procedures

Additional detail regarding the HPV assay would improve reproducibility, including its validation status, limit of detection, and any internal quality controls used. Please also clarify whether laboratory personnel were blinded to specimen type and whether quality assurance procedures were consistent across the participating countries.

3.Statistical Methods

The statistical analysis would benefit from clearer reporting. Please specify whether a sample size or power calculation was performed. Confidence intervals should be consistently reported for measures such as sensitivity, specificity, and agreement statistics. If not already included, stratified analyses by country or age group could help explore potential variability across sites.

4.Interpretation of Findings

The conclusions are generally supported by the results; however, where equivalence between sample types is implied, the language may need to be slightly moderated unless a predefined non-inferiority framework was applied. Expanding briefly on possible reasons for discordant results between specimen types would also strengthen the discussion.

5. Data Availability

The Data Availability Statement indicates that data are available upon reasonable request. Given PLOS ONE’s data-sharing policy, please clarify whether the dataset can be deposited in a public repository or provide a more detailed explanation of any restrictions that prevent open access.

Minor Comments

The manuscript is generally well written and clear, with only minor language refinements needed.

Please ensure consistent terminology (e.g., “first-void urine” throughout).

Tables should clearly specify denominators when percentages are reported.

Overall, this is an important and relevant study. With the clarifications above, the manuscript would be strengthened and better aligned with reporting standards.

Reviewer #10: The manuscript is well structured, all of the data was provided, statistical analysis was performed appropriately. the manuscript and data was supporting the conclusion and all of the required details were shared.

Reviewer #11: General Comments:

- This manuscript reports original, multicountry research comparing self-collected vaginal swabs (SCVS) and first-void urine (FVU) for HPV detection among young women in South Asia. The study addresses an important public health question in low- and middle-income countries and contributes valuable evidence for HPV surveillance and screening strategies.

- Minor language editing could further enhance readability.

Abstract

- The abstract is well structured and clearly summarizes the background, objectives, methods, key findings, and conclusions. The study design, sample size, laboratory methods, and main statistical approaches are appropriately described. The results are presented quantitatively and support the stated conclusions. The abstract accurately reflects the content of the manuscript and meets PLOS ONE standards for clarity and completeness

Introduction

- Provides a comprehensive overview of HPV epidemiology, cervical cancer burden, and screening challenges in South Asia. The authors effectively contextualize the need for self-sampling approaches and cite relevant international and regional literature. The rationale for comparing SCVS and FVU is well justified, and the research objectives are clearly stated.

- Lines 44–60 in the Introduction contain detailed country-specific descriptions of cervical cancer burden in Bangladesh, Pakistan, and Nepal. While this information is relevant, this section could be streamlined to improve conciseness and flow. Given that the study focuses on three countries, the authors may consider briefly explaining the rationale for selecting these specific settings (e.g., representativeness, programmatic relevance, epidemiological burden, or participation in the GLOBE-HPV project). In addition, the epidemiological background could be summarized more succinctly by presenting key indicators—such as HPV prevalence, cervical cancer incidence, and mortality rates—for each country, rather than providing extended narrative descriptions. Condensing this section would enhance readability while maintaining its contextual value, and would allow readers to more quickly understand the public health relevance of the selected study sites.

Materials and Methods

The manuscript states that in Bangladesh and Pakistan, participants were required to be married, whereas in Nepal eligibility was based on being sexually active regardless of marital status.

Could the authors please clarify the rationale for this difference in inclusion criteria across countries?

Specifically:

- Was marital status used as a proxy for sexual activity in Bangladesh and Pakistan due to sociocultural or ethical considerations?

- Were there regulatory or institutional constraints that prevented recruitment of unmarried sexually active women in those settings?

- How might this difference affect comparability of HPV prevalence and concordance estimates across the three countries?

A brief explanation in the Methods section, and possibly a note in the Limitations section addressing potential selection bias or differences in sexual behavior patterns, would strengthen transparency and interpretation of cross-country comparisons.

Sample Size

Could the authors please clarify:

- Whether a formal sample size or power calculation was performed for this concordance analysis?

- If so, which assumptions were used (expected HPV prevalence, anticipated agreement/Kappa value, precision, alpha level)?

- Whether the target of approximately 250 participants per country was based on logistical considerations, cohort design, or statistical requirements of the GLOBE-HPV project?

Results

- The manuscript reports no inadequate test results; however, additional clarification on data completeness (e.g., missing demographic or behavioral variables) would improve transparency.

- The description of discordant cases could be expanded, for example by indicating whether discordance was more frequent in specific subgroups (country, age range, or HPV type categories).

Discussion

The Discussion section is generally well written and provides an appropriate interpretation of the study findings in relation to existing literature. The authors successfully contextualize their results within prior studies on self-sampling for HPV detection and highlight the relevance for surveillance in low- and middle-income settings.

However, several aspects could be strengthened:

- Balance between interpretation and speculation Some explanations regarding differences in sensitivity and concordance between SCVS and FVU appear speculative and would benefit from stronger empirical support or clearer citation of supporting evidence.

- Cross-country comparability Given the differences in inclusion criteria and recruitment strategies across countries, the authors should more explicitly discuss how these variations may influence the comparability of findings and interpretation of pooled analyses.

- Public health and implementation implications While the potential use of SCVS and FVU in surveillance programs is mentioned, further discussion on operational feasibility, cost considerations, and integration into existing screening systems in South Asia would enhance the practical relevance of the findings.

- Limitations and potential biases The limitations section could be expanded to more clearly address potential selection bias, reporting bias related to self-sampling, and variability in laboratory processing across sites.

**********

6. PLOS authors have the option to publish the peer review history of their article (what does this mean?). If published, this will include your full peer review and any attached files.

If you choose “no”, your identity will remain anonymous but your review may still be made public.

**Do you want your identity to be public for this peer review?** For information about this choice, including consent withdrawal, please see our Privacy Policy.

Reviewer #1: No

Reviewer #2: No

Reviewer #3: **Yes:** Muhammad Saqib

Reviewer #4: **Yes:** Hussein Mussa Muafa

Reviewer #5: **Yes:** Ganesh Praneeth Roy Avula

Reviewer #6: No

Reviewer #7: **Yes:** Muhammad Saqib

Reviewer #8: No

Reviewer #9: No

Reviewer #10: **Yes:** Reenam Mushtaq

Reviewer #11: **Yes:** Jessica Marsigit

**********

[NOTE: If reviewer comments were submitted as an attachment file, they will be attached to this email and accessible via the submission site. Please log into your account, locate the manuscript record, and check for the action link "View Attachments". If this link does not appear, there are no attachment files.]

To ensure your figures meet our technical requirements, please review our figure guidelines: https://journals.plos.org/plosone/s/figures

You may also use PLOS’s free figure tool, NAAS, to help you prepare publication quality figures: https://journals.plos.org/plosone/s/figures#loc-tools-for-figure-preparation.

NAAS will assess whether your figures meet our technical requirements by comparing each figure against our figure specifications.

Attachment Submitted filename: Comments PLoS One.docx

Attachment Submitted filename: report_10.1101+2025.08.01.25332560.pdf

Attachment Submitted filename: General Comments.docx

# Author comment: Author response to Decision Letter 1

**DOI:** 10.1371/journal.pone.0350049.r002

29 Apr 2026

Authors’ response to reviewers' comments:

Reviewer #1

The article entitled, “Comparison of self-collected vaginal swabs and first-void urine for detection of human papillomavirus in sexually active girls and women in three South Asian countries,” by Telele et al., describes a cross-sectional study wherein the authors compared two non-invasive sampling methods for HPV DNA detection. The authors collected 753 samples from sexually active females ages 15–25 in Bangladesh, Pakistan, and Nepal using two methods, i.e., self-collected vaginal swabs (SCVS) and first-void urine (FVU). The authors used a harmonized protocol not only to assess HPV prevalence but also to determine concordance between the two sample types.

Though the study appears to be scientifically sound, and the manuscript is well organized and prepared, there are few changes required to strengthen the objectives and outcomes of the study. The manuscript can be accepted for publication in the PLoS One after replies to the following comments are addressed satisfactorily:

1. The authors cited similar studies that have compared self-collected vs urine samples for HPV. The authors should mention with clarity what is the uniqueness/novelty of the present study.

Response: While self-collected swabs and urine samples have been studied as methods for HPV detection, the methods used, including collection devices, preservatives, processing, extraction and assays all differ. Our results add to options that have been evaluated and include the ability to perform the collection and testing in a harmonized manner. Further, we included an exploration of the impact of viral load on the concordance between samples. We modified the introduction to clarify this (page 6, lines 98 – 101).

2. In the introduction section, instead of over emphasizing the HPV background and self-sampling the authors should focus on the research question and what knowledge gap is being filled by carrying out the present study.

Response: The introduction has been extensively modified to emphasize the knowledge gap being addressed.

3. The authors should provide the justification of cohort chosen especially the reason for age of 15-25 years and if / how it is related to HPV vaccination. Why do authors have not included broader age range? The reasoning behind the narrative should be provided in the manuscript as it would strengthen the manuscript.

Response: The cohorts were formed based on the aims of the GLOBE-HPV study. The age group for the comparison of sampling methods was selected primarily because of the likelihood of HPV acquisition. In addition, this age group is generally considered for “catch-up” vaccination, so providing an indication of exposure to vaccine types could be of importance to countries planning HPV vaccine campaigns.

4. The recruitment strategies detailed in the present study differed by country. Such as, the authors carried clinical based sampling in Nepal; demographic surveillance in Pakistan and sampling of married women in Bangladesh. The impact of recruitment strategy can be on the results / observations of the study. The authors should provide a clarification on the approach.

Response: Marital status was used as a proxy for sexual activity in Bangladesh and Pakistan due to sociocultural, religious, or ethical considerations. Asking about sexual activity outside of marriage is culturally sensitive and could cause repercussions for the participants. This explanation has been added. We do not think that this difference in recruitment impacts study findings as the cultures of Bangladesh and Pakistan result in near concordance between sexually active women and married women (page 9, lines 153 -156).

5. In discussion section, the authors could provide information on comparing the practical advantages between the two methods of sampling. They should also discuss how the present study outcomes can have influence on screening strategies. The authors can summarize the major findings/takeaways of the present study in conclusion.

Response: The major takeaway of the present study is included in the conclusion, that is, both sampling methods give similar HR-HPV results. We did not collect data on practical advantages between the two methods. These would include preference of the women (not addressed), costs of materials and disposal (different in each setting due to local importing suppliers and tariffs), and laboratory facilities (standardized and key assay instrument provided as part of GLOBE-HPV).

6. As the samples were self-collected, the authors should acknowledge the limitations of the method because of absence of clinician / trained personnel collected samples as it can impact the outcome of the result. The authors should mention other limitations too.

Response: The biggest limitation to the findings is that they apply to the specific sample collection tools, media, processing, extraction and assays used in this study and may not be generalizable to all methods. Lack of clinician collected samples as comparator was added to the limitations and the potential impact of population-specific factors and laboratory variations was added (page 20, lines 364 – 374).

7. The authors should provide the probable reasons for the observations that why Nepal had higher HPV prevalence than other two countries. Further, authors should mention if they have any plans for future studies to follow up or some other objectives.

Response: This study is focused on comparability of sampling methods. Discussion of country-specific differences in HPV prevalence as well as persistence will be addressed in the GLOBE-HPV publications.

8. The authors should ensure consistency in use of HPV-related terms. Such as at some places for High Risk ‘hr’ is used while at other places ‘HR’ is used. Authors should check the manuscript to make sure about uniformity.

Response: Checked.

9. Authors should make sure that all web references have accessed date and formatted as per PLoS One requirements. Also, make sure that all the references are cited and citations provided meet the PLoS One requirements.

Response: Checked

Reviewer #4:

This manuscript presents a valuable multi-country study evaluating the concordance between self-collected vaginal swabs (SCVS) and first-void urine (FVU) for HPV detection among young women in Bangladesh, Nepal, and Pakistan. The study addresses a critical public health need in resource-limited settings. The methodology is robust, the sample size is adequate, and the manuscript is well-written. The harmonized protocol across three countries is a significant strength.

However, I have a few minor comments and queries that, if addressed, would further strengthen the manuscript.

Specific Comments:

Sample Validity: The results state (Page 12, Line 196) that "No inadequate test results were reported from either sample type." Given the nature of self-collection and the large sample size (N=753 pairs), a 0% invalid rate is exceptionally evident of high-quality supervision or robust DNA extraction methods. Could the authors briefly clarify in the Methods section if any participants were excluded prior to the final N=753 count due to sample insufficiency, or if the "UCM" buffer and extraction protocol truly yielded 100% valid DNA for all collected samples? This detail speaks to the feasibility of this method in real-world surveillance.

Response: None of the samples were inadequate in this set of samples, although this has been encountered in the larger study. This information has been added to the results (page 13, lines 227).

Data Availability: The authors have indicated that data is available "upon reasonable request" due to ethical restrictions. Please ensure this complies with PLOS ONE’s strict data availability policy. If the data contains sensitive patient information that prevents public deposition, the specific ethical restrictions and the contact information for a non-author institutional body (e.g., an Ethics Committee) should be clearly stated in the final declaration, rather than just the authors' contact.

Response: De-identified individual participant data underlying the results reported in this article will be made available through Vivli (vivli.org), where access requires submission and approval of a formal data access request. This information has been added (page 1, lines 31-33).

Cycle Threshold (Ct) Analysis: The comparison of Ct values between concordant and discordant samples is a very insightful addition (Figure 1). It clearly demonstrates that discordance is driven by low viral loads. It would be beneficial to briefly discuss the clinical implication of this: are the "missed" infections in discordant cases likely to be clinically significant (e.g., persistent infections) or transient low-load infections?

Response: We do not have data to address the clinical significance of low viral load infections. While it is tempting to speculate that these are less likely to persist and progress, the study was not designed to address this question.

Demographics: The study population varies slightly by country (e.g., married women in Bangladesh/Pakistan vs. sexually active women in Nepal). While the authors acknowledge this in the limitations, a brief sentence in the Discussion on whether marital status (and implied sexual behavior differences) might impact the vaginal microbiome or viral load—and thus detection concordance—would be interesting, though not mandatory.

Response: These cohorts were intended to represent sexually active women, however asking about sexual activity outside of marriage in Bangladesh and Pakistan was not permissible due to sociocultural, religious, or ethical considerations. Marriage was used as a proxy for sexual activity. We do not think that this difference in recruitment impacts study findings as the cultures of Bangladesh and Pakistan result in near concordance between sexually active women and married women. The concordance findings between countries was not statistically significant. We cannot comment on potential impact of many variables (e.g. genital hygiene, douching, sexual history) that may impact HPV and vaginal microbiome.

Logistics: The conclusion emphasizes the potential for these methods in "resource-limited settings." However, the methods describe a cold chain (2-8^{\circ}C transport, -20^{\circ}C storage). In the Discussion, could the authors comment on the stability of samples in the UCM buffer at ambient temperature? This is crucial for true utility in remote areas without cold chain infrastructure.

Response: We did not include details about the cold chain, but this was achieved in remote areas with the use of cold packs. The manufacturer of the Colli-Pee device with preservative does indicate samples are stable at ambient temperature up to 7 days, however, as ambient temperatures could differ significantly between sites, we opted to provide consistency through the use of ice packs. Further work would be necessary to verify the time of stability of samples kept at ambient temperatures.

Conclusion:

The study is technically sound and provides important data for HPV surveillance strategies in South Asia. I recommend publication after these minor points are addressed.

Reviewer #6:

Introduction

* Your final aim statement says “sexually active girls and women aged 15–25 years” (line 85), but your Methods section indicates 18–25 in Pakistan and Nepal and “ever-married ≤25” in Bangladesh. Ensure the aim statement aligns with the enrolled age ranges ("18–25 years” overall, or “≤25 years"), maybe with a brief note about country-specific eligibility.

Response: In Bangladesh early age at marriage is a common practice, so married girls age 15 and older were included. The overall aim statement was intended to cover all three countries, with country-specific inclusion information provided in the methods.

* You have said that “Pakistan has not yet introduced the HPV vaccine” (lines 54–55). That is no longer accurate: Pakistan launched its first HPV vaccine rollout/campaign beginning September 15, 2025, with plans to integrate it into routine immunization thereafter.

Response: Thank you, we have added this good news to the introduction (page 5, lines 56-57).

* The reader will want a tighter argument for why concordance between SCVS and FVU matters in your specific context. I recommend adding 1–2 sentences on whether your primary use case is population-based surveillance, screening uptake, or post-vaccine monitoring, and why urine might be preferable in South Asia (acceptability, cultural norms, logistics, clinic capacity).

Response: We have revised the introduction and methods to clarify that this comparison was performed in the context of the GLOBE-HPV study, an epidemiologic study of HPV in multiple countries. The introduction states that urine sampling may be preferred over cervical or vaginal sampling in some cultures (page 6, lines 83-85).

* The introduction can be precise. Consider trimming 1–2 sentences on virology/classification, and use that space to sharpen the study rationale.

Response: The introduction has been sharpened and rationale clarified.

Methodology and Results

* The target populations and recruitment strategies vary (Bangladesh: “ever-married ≤25” with stratified systematic sampling; Pakistan: married 18–25; Nepal: sexually active 18–25). This affects both prevalence estimates and potentially concordance if underlying risk factors differ. Please provide baseline participant characteristics by country (age distribution, marital status, parity, education, contraceptive use, recent sexual activity, etc.) and discuss how these differences may influence outcomes.

Response: The data on participant characteristics and behaviors and their impact on HPV infection will be presented in subsequent GLOBE-HPV publications. The purpose of this analysis was to simply evaluate comparability of HPV detection by two sampling methods. As the concordance was high and similar across multiple settings, the information provides assurance that differences in epidemiologic characteristics and behaviors in these populations was not significant.

* Self-collection procedures require a little more detail, such as the order of collection and assistance/observation, FVU timing....

Response: We have added the instructions for sample collection given to participants to the supplemental materials. The order of sample collection was not specified, and this information is added to the methods (page 10, lines 166-167).

* The high overall agreement/negative agreement (Table 4) likely reflects low prevalence and a large proportion of negative pairs (as your negative agreement is >99%). This is expected but can overstate “equivalence.” Mention this issue in the discussion or emphasize positive concordance and directional discordance.

Response: It is correct that the negative agreement in low prevalence settings contributes to the high observed concordance. The data in Table 4 also show the lower positive concordance. This is a commonly used approach to compare qualitative (i.e. positive/negative) results between two methods. A statement has been added to the discussion to note this -- It should be noted that in settings with higher HPV prevalence, the positive agreement rates could be expected to be lower, affecting the overall agreement. (page 18, lines 309-310)

* Table 3 text: “full discordance ranged from 8.4% (38 HPV types)” appears to be a typo; it should likely be 28 HPV types.

Response: Yes, the text should read “full discordance ranged from 8.4% (28 types) to 0.7% (HPV 16/18)” but we corrected the typo (page 14, lines 248).

Reviewer #8

The inclusion criteria and clinical background of the study population are not clearly defined. It is not fully explained whether participants were attending for routine screening, symptomatic evaluation, abnormal cytology follow-up, or other clinical reasons. Because the sample is hospital-based, this may introduce selection bias and limit generalizability to the broader population. The authors should clearly describe the recruitment setting and clinical indications for participation.

Response: The study setting and rationale has been clarified in the introduction and study description.

Attachment Submitted filename: Response to Reviewers.docx

# Editor report: Decision Letter 1

**DOI:** 10.1371/journal.pone.0350049.r003

**Authors:** Ramokone Lebelo

7 May 2026

Comparison of self-collected vaginal swabs and first-void urine for detection of human papillomavirus in sexually active girls and women in three South Asian countries

PONE-D-25-43925R1

Dear Dr. Telele

We’re pleased to inform you that your manuscript has been judged scientifically suitable for publication and will be formally accepted for publication once it meets all outstanding technical requirements.

Within one week, you’ll receive an e-mail detailing the required amendments. When these have been addressed, you’ll receive a formal acceptance letter and your manuscript will be scheduled for publication.

An invoice will be generated when your article is formally accepted. Please note, if your institution has a publishing partnership with PLOS and your article meets the relevant criteria, all or part of your publication costs will be covered. Please make sure your user information is up-to-date by logging into Editorial Manager at Editorial Manager® and clicking the ‘Update My Information' link at the top of the page. For questions related to billing, please contact billing support.

If your institution or institutions have a press office, please notify them about your upcoming paper to help maximize its impact. If they’ll be preparing press materials, please inform our press team as soon as possible -- no later than 48 hours after receiving the formal acceptance. Your manuscript will remain under strict press embargo until 2 pm Eastern Time on the date of publication. For more information, please contact onepress@plos.org.

Kind regards,

Ramokone Lisbeth Lebelo, Ph.D

Academic Editor

PLOS One

# Editor report: Acceptance letter

**DOI:** 10.1371/journal.pone.0350049.r004

**Authors:** Ramokone Lebelo

PONE-D-25-43925R1

PLOS One

Dear Dr. Telele,

I'm pleased to inform you that your manuscript has been deemed suitable for publication in PLOS One. Congratulations! Your manuscript is now being handed over to our production team.

At this stage, our production department will prepare your paper for publication. This includes ensuring the following:

* All references, tables, and figures are properly cited

* All relevant supporting information is included in the manuscript submission,

* There are no issues that prevent the paper from being properly typeset

You will receive further instructions from the production team, including instructions on how to review your proof when it is ready. Please keep in mind that we are working through a large volume of accepted articles, so please give us a few days to review your paper and let you know the next and final steps.

Lastly, if your institution or institutions have a press office, please let them know about your upcoming paper now to help maximize its impact. If they'll be preparing press materials, please inform our press team within the next 48 hours. Your manuscript will remain under strict press embargo until 2 pm Eastern Time on the date of publication. For more information, please contact onepress@plos.org.

You will receive an invoice from PLOS for your publication fee after your manuscript has reached the completed accept phase. If you receive an email requesting payment before acceptance or for any other service, this may be a phishing scheme. Learn how to identify phishing emails and protect your accounts at https://explore.plos.org/phishing.

If we can help with anything else, please email us at customercare@plos.org.

Thank you for submitting your work to PLOS ONE and supporting open access.

Kind regards,

PLOS ONE Editorial Office Staff

on behalf of

Dr. Ramokone Lisbeth Lebelo

Academic Editor

PLOS One
